# Comparative analysis of complete *Artemisia* subgenus *Seriphidium* (Asteraceae: Anthemideae) chloroplast genomes: insights into structural divergence and phylogenetic relationships

**DOI:** 10.1186/s12870-023-04113-1

**Published:** 2023-03-10

**Authors:** Guangzhao Jin, Wenjun Li, Feng Song, Lei Yang, Zhibin Wen, Ying Feng

**Affiliations:** 1 State Key Laboratory of Desert and Oasis Ecology, Xinjiang Institute of Ecology and Geography, Chinese Academy of Sciences, Urumqi, 830011 China; 2 The Herbarium of Xinjiang Institute of Ecology and Geography, Chinese Academy of Sciences, Urumqi, 830011 China; 3 University of Chinese Academy of Sciences, Beijing, 100094 China; 4 Key Laboratory of Plant Resources Conservation and Sustainable Utilization, South China Botanical Garden, Chinese Academy of Sciences, Guangzhou, 510650 China

**Keywords:** Compositae, Comparative genomics, Molecular markers, Phylogenomics

## Abstract

**Background:**

*Artemisia* subg. *Seriphidium*, one of the most species-diverse groups within *Artemisia*, grows mainly in arid or semi-arid regions in temperate climates. Some members have considerable medicinal, ecological, and economic value. Previous studies on this subgenus have been limited by a dearth of genetic information and inadequate sampling, hampering our understanding of their phylogenetics and evolutionary history. We therefore sequenced and compared the chloroplast genomes of this subgenus, and evaluated their phylogenetic relationships.

**Results:**

We newly sequenced 18 chloroplast genomes of 16 subg. *Seriphidium* species and compared them with one previously published taxon. The chloroplast genomes, at 150,586–151,256 bp in length, comprised 133 genes, including 87 protein-coding genes, 37 tRNA genes, 8 rRNA genes, and one pseudogene, with GC content of 37.40–37.46%. Comparative analysis showed that genomic structures and gene order were relatively conserved, with only some variation in IR borders. A total of 2203 repeats (1385 SSRs and 818 LDRs) and 8 highly variable loci (*trnK* – *rps16*, *trnE* – *ropB*, *trnT*, *ndhC* – *trnV*, *ndhF*, *rpl32* – *trnL*, *ndhG* – *ndhI* and *ycf1*) were detected in subg. *Seriphidium* chloroplast genomes. Phylogenetic analysis of the whole chloroplast genomes based on maximum likelihood and Bayesian inference analyses resolved subg. *Seriphidium* as polyphyletic, and segregated into two main clades, with the monospecific sect. *Minchunensa* embedded within sect. *Seriphidium*, suggesting that the whole chloroplast genomes can be used as molecular markers to infer the interspecific relationship of subg. *Seriphidium* taxa.

**Conclusion:**

Our findings reveal inconsistencies between the molecular phylogeny and traditional taxonomy of the subg. *Seriphidium* and provide new insights into the evolutionary development of this complex taxon. Meanwhile, the whole chloroplast genomes with sufficiently polymorphic can be used as superbarcodes to resolve interspecific relationships in subg. *Seriphidium*.

**Supplementary Information:**

The online version contains supplementary material available at 10.1186/s12870-023-04113-1.

## Background

The genus *Artemisia* L., comprising ca. 500 herb and shrub species, is one of the largest in the Asteraceae [1–5]. Members of this genus are distributed mainly in temperate regions of the northern hemisphere [1, 6], with the current centers of species diversity located in China and surrounding areas followed by Russia and adjacent states, Europe, Americas and North Africa [7–9]. *Artemisia* typically attracts extensive scientific interest because of its antimalarial properties, and other pharmacological and economic value [1, 10, 11]. Although *Artemisia* is currently divided into the generally accepted five subgenera [subg. *Artemisia*, subg. *Absinthium* (Miller) Less., subg. *Dracunculus* (Besser) Rydb., subg. *Tridentatae* (Rydb.) McArthur. and subg. *Seriphidium* Besser ex Less] based on morphological and molecular data [12–18], there has been some controversy about its definition and infrageneric delimitation.

The subg. *Seriphidium*, one of the most diverse taxa in *Artemisia* [18], comprises ca. 130 species and 30 infraspecific taxa worldwide [3, 9, 19]. This subgenus grows mainly in arid and semi-arid regions of Central Asia and Northwest China, with a few species spreading to the Middle East, North Africa and Europe [19]. Its species are usually drought-, cold- and salinity-tolerant, and can become dominant in arid and semi-arid areas, playing an important ecological role in terms of wind and sand control [20]. In addition, some are rich in essential oils and terpenes, having anti-malarial, anticancer and antidiabetic properties [7, 20–22]. However, the gaps that remain in our knowledge of the subg. *Seriphidium* and of its taxonomic complexity still call for further research. Since Besser grouped all homogamous species of *Artemisia* in sect. *Seriphidium* Besser [23, 24], the first comprehensive revision of *Seriphidium* taxa was not published until 1961, Poljakov separated the homogamous species from *Artemisia* in Eurasia and established the new genus *Seriphidium* (Besser) Poljakov [25]. However, the same author did not follow his own proposal in *Flora of the USSR* published the same year and still treated *Seriphidium* as a subgenus within *Artemisia* [7], and divided the subg. *Seriphidium* into two sections: (i) sect. *Seriphidium* with pinnate-lobed leaves; and (ii) sect. *Junceum* with mostly 3-lobed lower stem leaves. After studying *Seriphidium* in Eurasia and North Africa, Filatova in 1986 proposed a different proposal from Poljakov’s on the two sections within subg. *Seriphidium*, dividing the subgenus into six sections [sect. *Calciphilum*, sect. *Junceum*, sect. *Leucophyton*, sect. *Sclerophyllum*, sect. *Halophilum* and sect. *Pycnanthum*] based on traits such as leaf type, leaf segments, involucre and florets [26].

When Ling studied the entire genus *Artemisia* and its allies [9, 19, 20, 27], he supported the taxonomic view of establishing *Seriphidium* as an independent genus based on homogamous flowers, involucral bracts multilayered and flowering pattern, and divided the 130 *Seriphidium* taxa (containing species and infraspecific taxa) into three sections: (i) sect. *Seriphidium* with pinnate-lobed leaves; (ii) sect. *Junceum* with mostly 3-lobed lower stem leaves; and (iii) sect. *Minchunensa* with pectinate or narrowly serrate pinnatisect leaves. The first two sections are similar in species composition to the two sections within subg. *Seriphidium* established by Poljakov. Moreover, sect. *Junceum* (*A. juncea*) and sect. *Minchunensa* (*A. minchunensis*) are both monospecific groups. However, the rationality of the classification of subg. *Seriphidium* based on morphological traits remains to be further explored.

In the past two decades, the emergence of molecular systematics has provided new methods for studying the systematic relationships between complex taxa [28]. Some molecular markers from both the nuclear and plastid genomes, including nuclear ribosomal DNA internal and external transcribed spacers (ITS and ETS) and chloroplast fragments (*matK*, *rbcL*, *rpl32* – *trnL*, *ndhC* – *trnV* and *psbA* – *trnH*) have been used to estimate phylogenetic relationships within *Artemisia* [4, 5, 12–18, 29–32]. Unfortunately, the subg. *Seriphidium* has received less attention in comparison to other subgenera of *Artemisia* [18]. Furthermore, many of the prior phylogenetic studies of subg. *Seriphidium* [4, 18, 31], based on plastid or nuclear gene fragments, have achieved low resolution at major clade nodes, owing to the high sequence similarity between its closely related taxa arising from its rapid evolutionary radiation and hybridization. Recent molecular phylogenies did not support the traditional morphology-based subg. *Seriphidium* classifications, have revealed that it is not monophyletic [18]. At present, phylogenetic relationships among the major lineages of the subg. *Seriphidium* remain uncertain, such as owing to limited sampling, the systematic position of the Chinese endemic species *A. minchunensis* which constitutes the monospecific group (sect. *Minchunensa*) has not been clarified. Further investigations, based on a combination of representative sampling and sequences with rich genetic information, is therefore necessary to reconstruct these phylogenetic relationships.

The chloroplast, a multifunctional plant organelle, plays an important role in photosynthesis as well as various metabolic processes [33–35]. In most angiosperms, the complete chloroplast genome is usually a double-stranded, circular and quadripartite structure, consisting of four evolutionarily relatively conserved regions: a large single copy region (LSC), a small single copy region (SSC) and a pair of inverted repeat regions (IRa and IRb) [36–38]. Compared to plant mitochondrial and nuclear genomes, the chloroplast genomes of most land plants exhibit slow evolution and uniparental inheritance, and are appropriately sized and relatively conservative in structure [21, 39, 40]. Unlike gene fragments, complete chloroplast genome contains much genetic information and many mutation sites, contributing to resolving the complex evolutionary relationships in land plants [41]. The complete chloroplast genome is therefore widely used for phylogenetic inference and species delimitation, such as *Ligularia* (Asteraceae) [42], *Amomum* (Zingiberaceae) [43], *Calligonum* (*Polygonaceae*) [44], *Ilex* (Aquifoliaceae) [45] and *Rhododendron* (Ericaceae) [46]. It is worth noting that a recent study analyzed 18 *Artemisia* species from East Asia using the whole chloroplast genome, and the results showed that whole chloroplast genomes with sufficient polymorphic genetic information loci could be used to resolve interspecific relationships within *Artemisia* [47]. Unfortunately, this study did not include any subg. *Seriphidium* species. Nevertheless, this provides a reference for exploring the use of whole chloroplast genomes for resolving the systematic position and interspecific relationships of taxa in subg. *Seriphidium*.

To date, GenBank (National Center for Biotechnology Information; accessed 1 April 2022) contains the complete chloroplast genome for only one species (*A. maritima*) of subg. *Seriphidium*, accounting for ca. 1% of its species diversity. Based on the above problems of subg. *Seriphidium*, here we newly sequenced 18 complete chloroplast genomes from 16 subg. *Seriphidium* species, collected in arid and semi-arid regions of northwestern China and adjacent countries (Russia and Tajikistan). It is noteworthy that these samples have included representative species from three sections within subg. *Seriphidium* with reference to Ling (1991) [19], particularly *A. minchunensis* which constitutes the monospecific group (sect. *Minchunensa*). The main objectives of the present study were: (1) to examine variation in the structure and composition of subg. *Seriphidium* chloroplast genomes; (2) to assess the ability of the complete chloroplast genome to resolve interspecific relationships within this subgenus, and (3) to explore the systematic position of the main subg. *Seriphidium* taxa, especially *A. minchunensis*. This study provides guidance for the taxonomic revision of the entire subg. *Seriphidium*, and facilitates the development and utilization of its genetic resources.

## Results

### Subg. *Seriphidium* chloroplast genome structural variation

**Fig. 1:**
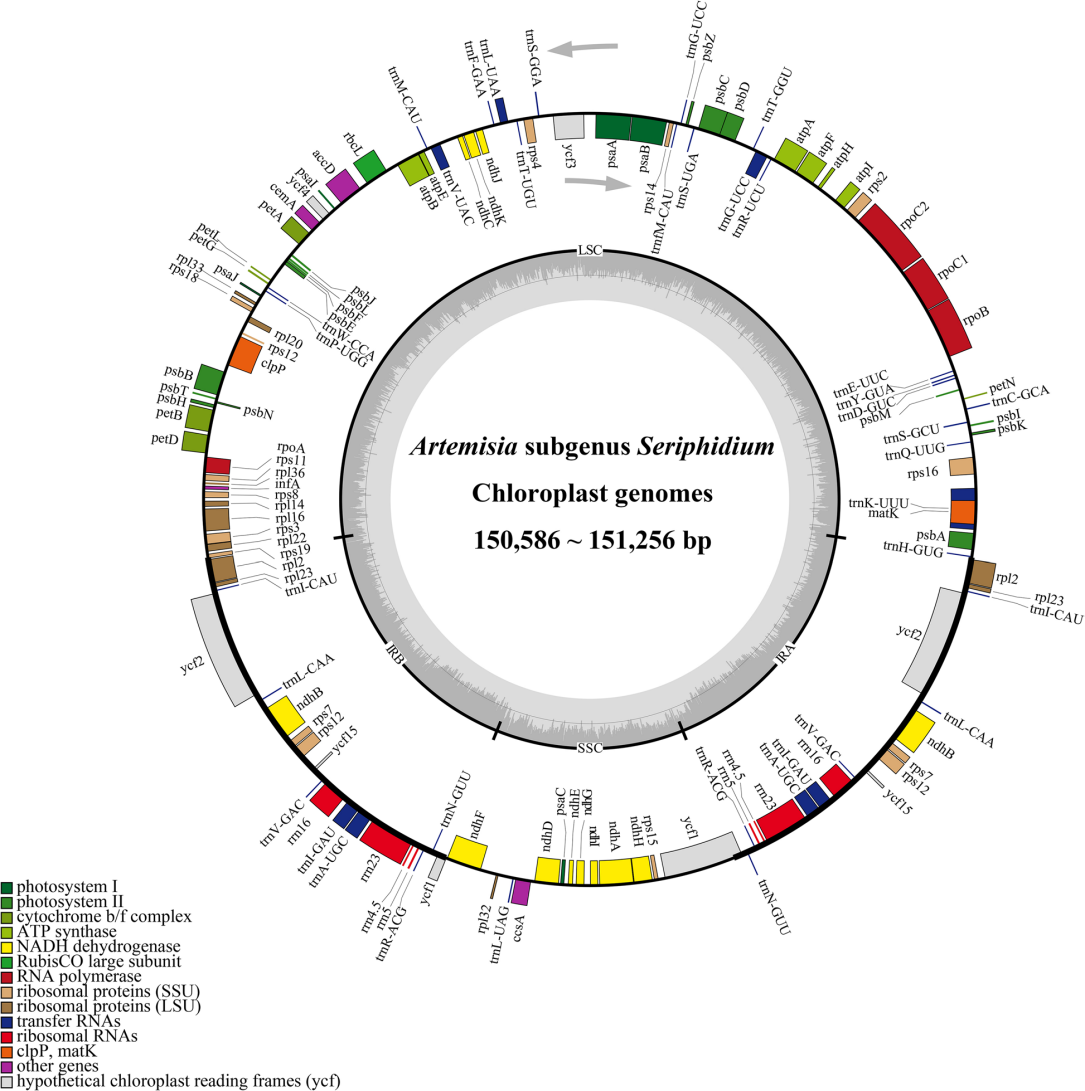
Gene circle map of 16 newly sequenced *Artemisia* subg. *Seriphidium* species. Arrows indicate transcription direction. Genes located outside the outer circle were transcribed counter-clockwise, and those inside were transcribed clockwise. Colored bars indicate different functional groups. Thick lines of the large circle indicate the extent of inverted repeat regions (IRa and IRb) that separate the genome into large single and small copy regions (LSC and SSC, respectively). Darker gray columns in the inner circle correspond to guanine-cytosine content, and light gray to adenosine-thymine content

All of the 18 newly sequenced subg. *Seriphidium* chloroplast genomes possessed the typical vascular plant quadripartite structure, comprising LSC, SSC, IRa and IRb regions (Fig. 1). Genome length ranged from 150,586 bp (*A. ferganensis*) to 151,256 bp (*A. santonicum*). LSC region length ranged from 82,313 bp (*A. ferganensis*) to 82,976 bp (*A. santonicum*). SSC region length ranged from 18,329 bp (*A. ferganensis*) to 18,379 bp (*A. santolina*). IR region length ranged from 24,959 bp (*A. sawanensis* and *A. schrenkiana*) to 24,972 bp (*A. ferganensis*) (Table 1). Interestingly, while *A. ferganensis* had the shortest total chloroplast genome, and shortest LSC and SSC regions, it had the longest inverted repeat regions. There was slight variation in guanine-cytosine contents, at 37.40 to 37.46% (Table 1). All 18 plastomes contained 87 protein-coding genes, 37 transfer RNA (tRNA) genes, 8 ribosomal RNA (rRNA) genes, and one pseudogene, and exhibited the same order and orientation of syntenic blocks (Table 1; Additional file 1: Table S2; Additional file 2: Fig. S1), indicating that these chloroplast genomes are highly conserved and collinear.

**Table 1:** Summary of complete chloroplast genomes of 16 newly sequenced *Artemisia* subg. *Seriphidium* species

Taxon	Sample ID	Herbarium /Voucher No.	Localities	Locations		Accession number	Gene number			Length (bp)				GC(%)
				N	E		CDS	tRNA	rRNA	Total	LSC	IRs	SSC	
*A. ferganensis*	J-3	XJBI/jgz-3	China: Xinjiang	39.535	75.614	ON871797	87	37	8	150,586	82,313	24,972	18,329	37.46
*A. finita*	J-23	PE/8554	China: Neimenggu	–	–	ON871798	87	37	8	151,157	82,868	24,969	18,351	37.46
*A. juncea*	J-5-1	XJBI/jgz-18-1	China: Xinjiang	43.867	87.564	ON871799	87	37	8	151,004	82,749	24,962	18,331	37.46
*A. juncea*	J-5-2	XJBI/jgz-18-2	China: Xinjiang	43.867	87.564	ON871800	87	37	8	151,004	82,749	24,962	18,331	37.46
*A. karatavica*	J-25	PE/1343386	Russia	–	–	ON871801	87	37	8	151,114	82,806	24,969	18,370	37.44
*A. kaschgarica*	J-4	XJBI/jgz-045–2	China: Xinjiang	44.326	85.518	OL890688	87	37	8	151,091	82,808	24,969	18,345	37.44
*A. lercheana*	J-40	PE/1341747	Russia	–	–	ON871802	87	37	8	151,089	82,801	24,969	18,350	37.45
*A. leucotricha*	J-42	PE/1342944	Tadzhikistan	–	–	ON871803	87	37	8	151,056	82,774	24,968	18,346	37.46
*A. minchunensis*	J-2-1	XJBI/jgz-103	China: Gansu	38.981	103.548	ON871804	87	37	8	151,099	82,810	24,970	18,349	37.45
*A. minchunensis*	J-2-1	XJBI/jgz-104	China: Gansu	38.981	103.548	ON871805	87	37	8	151,099	82,810	24,970	18,349	37.45
*A. santolina*	J-6	XJBI/jgz-052	China: Xinjiang	44.483	82.911	ON871806	87	37	8	151,112	82,795	24,969	18,379	37.44
*A. santonicum*	J-33	PE/1339064	Russia	–	–	ON871807	87	37	8	151,256	82,976	24,969	18,342	37.40
*A. sawanensis*	J-7-5	XJBI/jgz-086-3	China: Xinjiang	47.499	87.736	ON871808	87	37	8	151,066	82,799	24,959	18,349	37.45
*A. schrenkiana*	J-7-4	XJBI/jgz-086-1	China: Xinjiang	47.499	87.736	ON871809	87	37	8	151,073	82,806	24,959	18,349	37.45
*A. scopaeformis*	J-26	XJBI/jgz-43-1	China: Xinjiang	42.996	88.749	ON871810	87	37	8	151,017	82,727	24,970	18,350	37.46
*A. sublessingiana*	J-9	XJBI/jgz-080-1	China: Xinjiang	47.559	86.909	ON871811	87	37	8	151,065	82,787	24,969	18,340	37.45
*A. terrae-albae*	J-10	XJBI/jgz-079-1	China: Xinjiang	47.451	86.815	ON871812	87	37	8	151,107	82,824	24,969	18,345	37.45
*A. transiliensis*	J-13	XJBI/jgz-012-3	China: Xinjiang	43.534	87.167	ON871813	87	37	8	151,112	82,832	24,970	18,350	37.44

*CDS* coding sequence, *IRs* inverted repeat regions, *tRNA* transfer RNA, *SSRs* Simple sequence repeats, *LSC* large single copy, *SSC* small single copy, *GC* guanine-cytosine, *N* north latitude, *E* east longitude

### IR expansion and contraction

**Fig. 2:**
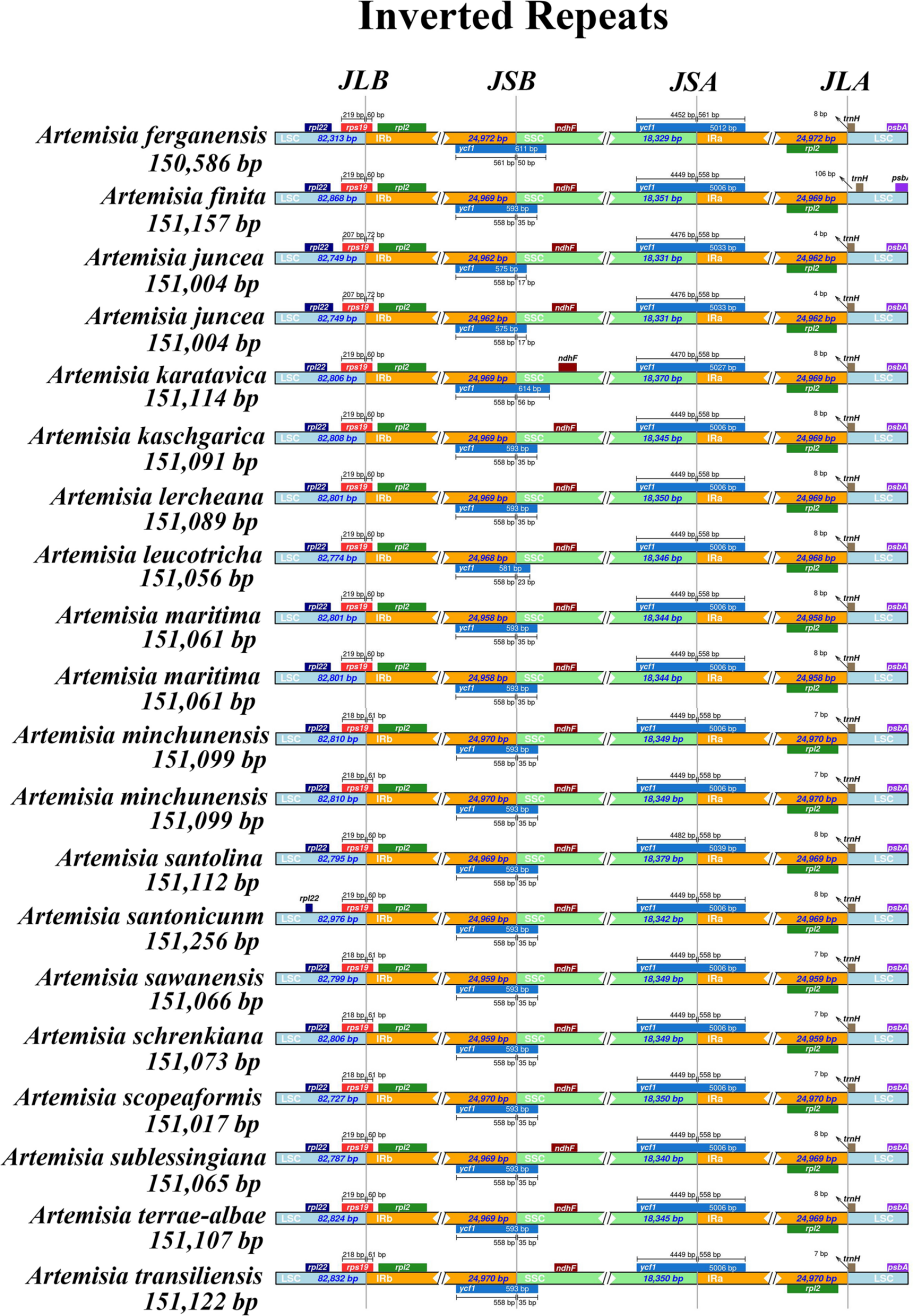
Comparison of the single copy-inverted repeat junctions among the 17 subg. *Seriphidium* species (16 newly sequenced and one published [21]). JLB, JSB, JSA and JLA: LSC/IRb, SSC/IRb, SSC/IRa and LSC/IRa, respectively. IRa, IRb: two IR regions that are identical but in opposite orientations; LSC: large single copy; SSC: small single copy

Comparative sequence analysis of 17 subg. *Seriphidium* species (16 newly sequenced and one published [21]) revealed that chloroplast genome structure and gene order were highly conserved, although with slight variations at the IR boundaries (Fig. 2). The length of IR was relatively consistent among all subg. *Seriphidium* species. *A. sawanensis* and *A. schrenkiana* had the shortest IR length (24,959 bp), while *A. ferganensis* had the longest (24,972 bp). All of the subg. *Seriphidium* chloroplast genomes had LSC/IRb junctions in gene *rps19*, with 60 to 72 bp crossing into the IRb region, indicating an expansion of the IR in these species (Fig. 2). Similarly, in all of subg. *Seriphidium* chloroplast genomes, the IRb/SSC junctions were located in gene *ycf1*, extending 17–35 bp into the SSC region, away from the *ndhF* gene. All of the subg. *Seriphidium* chloroplast genomes had SSC/IRa junctions located in gene *ycf1*, extending 561–558 bp into the IRa region. Most of the IRa/LSC junctions were located between genes *rpl2* and *trnH*, with 4–8 bp far from the gene *trnH*, although in *A. finite*, the IRa/LSC junction was located 106 bp far from gene *trnH* (Fig. 2).

### Analysis of repeats

Simple sequence repeats (SSRs) are shorter tandem repeats consisting of 1–6 bp repeat units and are also known as microsatellite repeats. In total, 1385 SSRs were detected in the 20 subg. *Seriphidium* chloroplast genomes (17 species), including 777 mononucleotides (mono-), 216 dinucleotides (di-), 78 trinucleotides (tri-), 275 tetranucleotides (tetra-), 38 pentanucleotides (penta-), and one hexanucleotide (hex-) (Fig. S2a; Additional file 1: Table S3). Most of the SSRs were located in LSC regions (1088), followed by SSCs (181) and IR (116) regions (Fig. S2b; Additional file 1: Table S3). Moreover, these SSRs were mainly distributed in intergenic spacer regions (IGS) (1017), with some in CDS (227) and intron regions (141) (Fig. S2c; Additional file 1: Table S3). Among the mononucleotide repeats, A/T repeats were most frequent; C repeats were present in all but two taxa (*A. ferganensis* and *A. maritima*); and no G repeats were detected (Fig. S2d; Additional file 1: Table S3). Dinucleotide repeats were represented by only the AT/TA motif. Trinucleotide repeats (ATT/TTC) were present in all 20 subg. *Seriphidium* chloroplast genomes analyzed, however only one trinucleotide repeat (AAT) was detected in *A. finite*. Tetra- and pentanucleotide contained motifs AATA/AATC, AAAT/AATT, ATTG/CAAT, ATTT/TAAT, TATT/TTTC and TTAA/TTTA, as well as AAATT/ACGAC, ATAAA/ATATT, ATTTA/TATAT, and TTAAT repeats, respectively. Furthermore, only one hexanucleotide (AATATA) was detected distributed in LSC region of *A. finita* (Fig. S2d; Additional file 1: Table S3).

**Fig. 3:**
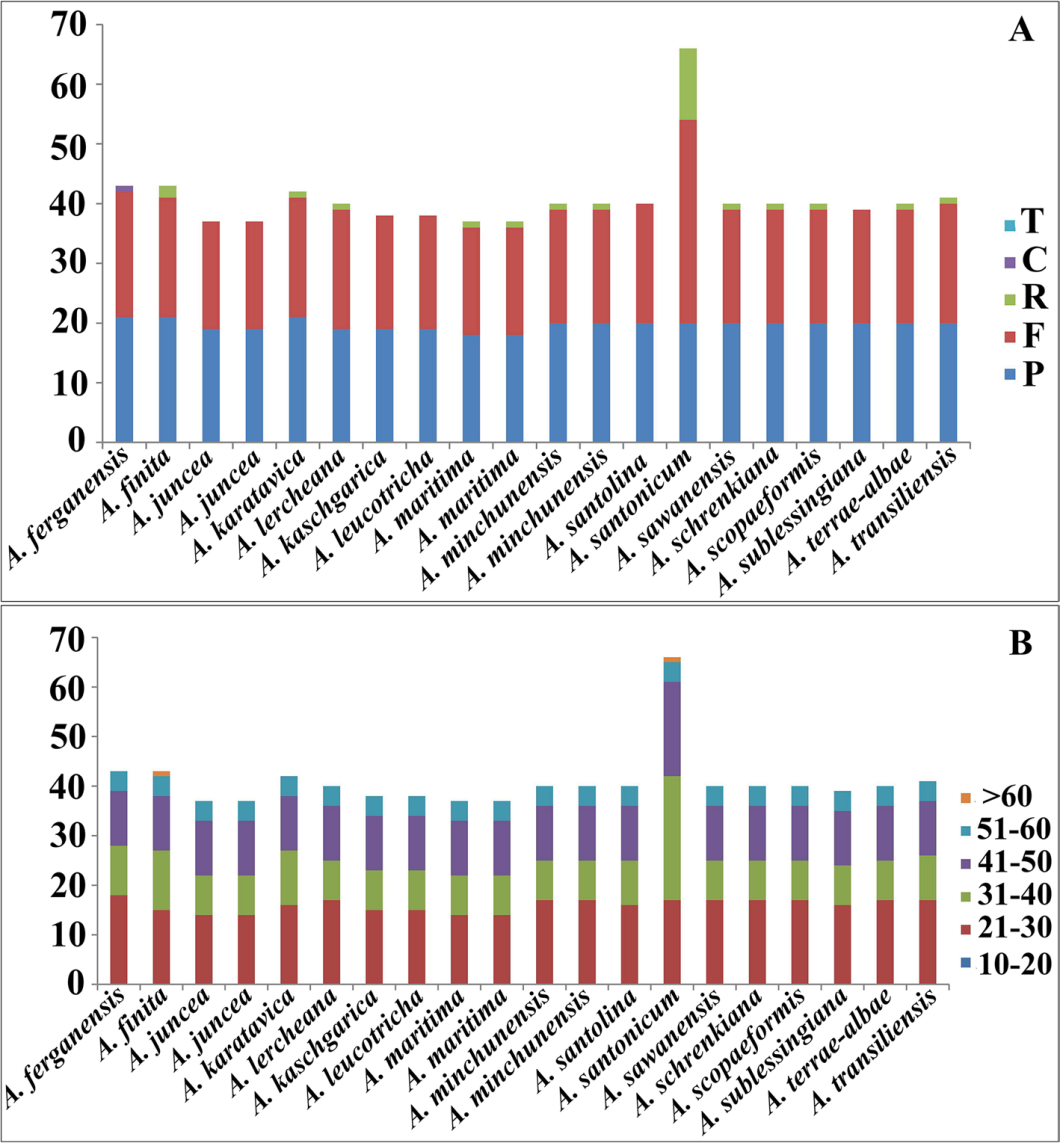
Long dispersed repeats of 20 *Artemisia* subg. *Seriphidium* chloroplast genomes. **A** Numbers of the five long repeat types; **B** Long dispersed repeat size

The forward (F), palindromic (P), reverse (R), and complement (C) repeat sequences in the 20 subg. *Seriphidium* chloroplast genomes (17 species) were detected using REPuter. In total, 818 long dispersed repeats were detected, including 398 forward, 394 palindromic, 25 reverse and one complement repeats (Additional file 1: Table S4). All species had forward and palindromic repeats, only one complement repeat was detected, in *A. ferganensis*. Approximately half (12/20) of the species had 1 or 2 reverses. Interestingly *A. santonicum* had 12 reverses, far more than the other species (Fig. 3A; Additional file 1: Table S4). Long dispersed repeat length was variable, at 30–86 bp, most commonly 30–50 bp. However, there were only two repeat regions were > 60 bp long [*A. finita* (86 bp) and *A. santonicum* (85 bp)] (Fig. 3B; Additional file 1: Table S4).

### Hypervariable regions and genomic divergence

Nucleotide variability (Pi) was 0.000–0.00557 (average, 0.00115) for the 18 newly assembled plastomes and two *A. maritima* plastomes from GenBank (MK532038 and NC_045093). At the cutoff value of Pi > 0.0045, eight highly variable regions were identified: *trnK-UUU* – *rps16*, *trnE-UUC* – *ropB*, 35 bp + *trnT-GGU* + 508 bp, *ndhC* – *trnV-UAC*, 123 bp + *ndhF*, *rpl32* – *trnL-UAG*, *ndhG* – *ndhI* and *ycf1*(1010–4275 bp) (Fig. S3; Additional file 1: Table S5). Four of these (*trnK-UUU* – *rps16*, *trnE-UUC* – *ropB*, 35 bp + *trnT-GGU* + 508 bp and *ndhC* – *trnV-UAC*) are located in the LSC region; while the other four are in the SSC region (Fig. S3). For these hypervariable loci, Pi ranges from 0.00451 (*ndhC* – *trnV-UAC*) to 0.00557 (*ndhG* – *ndhI*) (Additional file 1: Table S5).

The results of the sequence identity analysis of the 20 subg. *Seriphidium* chloroplast genomes (17 species), with *A. ferganensis* chloroplast genome as reference (Additional file 2: Fig. S4), are consistent with those of the nucleotide diversity analysis: IR regions were more conserved than SC regions, and non-coding regions were more divergent than coding regions. For the 20 chloroplast genomes, the divergent regions were in IGS, such as *trnE-UUC* – *ropB*, *trnS-GGA* – *ycf3*, *trnV-UAC* – *ndhC*, *psbE* – *petL*, *rbcL* – *accD*, *petA* – *psbJ* and *rpl32* – *trnL-UAG*. One distinct gap was observed, in the *psbM* region of the *A. sawanensis* chloroplast genome (Additional file 2: Fig. S4). In total, 931 polymorphic sites, 273 singleton variable sites, and 658 parsimony informative sites were detected among the 20 chloroplast genome sequences.

### Molecular markers for subg. *Seriphidium* species

To explore subg. *Seriphidium* molecular markers with increased resolution of phylogeny reconstruction, we tested eight screened highly variable regions and their combinations. Comparative sequence analysis revealed that *ndhF* is highly polymorphic in the subg. *Seriphidium* plastomes (Table 2). We constructed phylogenetic trees for each of the eight highly variable regions screened from whole chloroplast genes using 17 subg. *Seriphidium* species (16 newly sequenced and one published [21]) and assessed their potential potency. Our results revealed that the resolution of phylogenetic trees constructed based on each highly variable region was low (Additional file 2: Fig. S5–12). Moreover, the resolution of phylogenetic tree constructed using tandem sequences from eight highly variable regions was improved for the major clades compared to each highly variable region, but there are still deficiencies in discriminating at interspecific relationship (Additional file 2: Fig. S13). To further explore the resolution of phylogenetic tree, we made a first attempt to use whole chloroplast genome for 17 subg. *Seriphidium* species (16 newly sequenced and one published). We found that the resolution of phylogenetic tree was extremely high, both in the major clades and among species (Additional file 2: Fig. S14).

**Table 2:** DNA polymorphisms identified in 17 *Artemisia* subg. *Seriphidium* species (16 newly sequenced and one published)

Loci	Length (bp)	Number of sequence	Polymorphic site	Singleton variable sites	Parsimony informative sites
*ndhC* – *trnV-UAC*	1180	20	17	3	14
*trnE-UUC* – *ropB*	877	20	12	3	9
*trnK-UUU* – *rps16*	855	20	25	9	16
*rpl32* – *trnL-UAG*	923	20	13	2	11
*ndhG* – *ndhI*	376	20	16	8	8
*trnT-GGU*	68	20	2	1	1
*ndhF*	2234	20	443	437	6
*ycf1*	5073	20	72	21	51

### Phylogenetic analysis

**Fig. 4:**
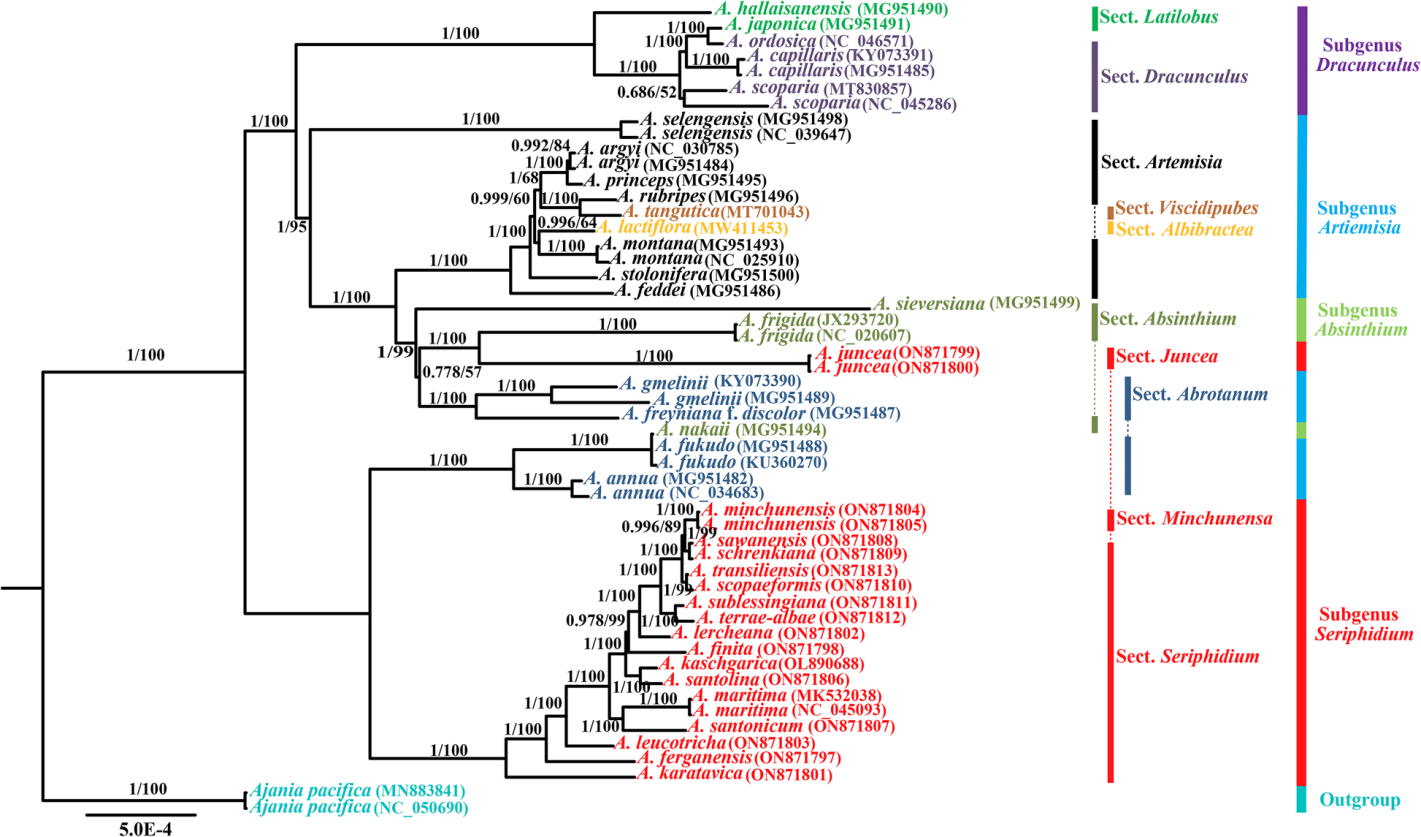
Phylogenetic tree inferred from Bayesian inference (BI) and maximum likelihood (ML) analyses, using the complete chloroplast genomes of 38 *Artemisia* species. Branch lengths were estimated using Bayesian inference. Numbers near the nodes are Bayesian posterior probabilities (to the left) and maximum likelihood bootstrap support values (to the right). Colored lines and braces at the right indicate the traditional section and subgenus classification of *Artemisia*. The sections of *Artemisia* subg. *Seriphidium* are divided according to Ling (1991) [19]

To evaluate the monophyly of subg. *Seriphidium* and its phylogenetic relationship with other subgenus in *Artemisia*, we reconstructed phylogenetic relationships based on 52 complete chloroplast genomes and 80 protein-coding genes from 38 *Artemisia* species, using Bayesian inference (BI) and maximum likelihood (ML), with the closely related species *Ajania pacifica* (NC_050690 and MN883841) as outgroup. The total alignment length (after removing one inverted repeat) was 125,171 bp, with 849 singleton variable sites and 1707 parsimony informative sites. The backbones of the BI and ML trees were nearly identical, whether based on complete chloroplast genomes or protein-coding genes, hence we present only the tree (branch lengths were estimated by BI analysis) for the whole chloroplast genome with posterior probability (PP) and bootstrap support (BS) values shown (Fig. 4; BI PP: 1.00; ML BS: 100%).

Based on these phylogenetic analyses, *Artemisia* is monophyletic; most of the clades have high support, with all samples of the same species clustered together (Fig. 4). All individuals of subg. *Dracunculus* are clustered together in a monophyletic clade (BI PP: 1.00; ML BS: 100%), but neither sect. *Latilobus* nor sect. *Dracunculus* within subg. *Dracunculus* are monophyletic. With the exception of sect. *Viscidipubes* and sect. *Albibractea*, the subg. *Artemisia* and its two other sections (sect. *Artemsia* and sect. *Abrotanum*) were recovered as polyphyletic (Fig. 4). Subg. *Absinthium*, with only one sect. *Absinthium*, was resolved as polyphyletic as well. Subg. *Seriphidium* is fully nested within genus *Artemisia*, forming two highly supported clades (Fig. 4; BI PP: 1.00; ML BS: 100%). Within subg. *Seriphidium*, a small clade containing *A. juncea* (sect. *Juncea*) forms a sister group to *A. frigida* (sect. *Absinthium*), and is located far from the other large monophyletic clade consisting of sect. *Seriphidium* and sect. *Minchunensa*. However, the inclusion of sect. *Minchunensa* within sect. *Seriphidium* is unexpected.

## Discussion

### Comparison of subg. *Seriphidium* chloroplast genomes

As in most angiosperms [36], we found that subg. *Seriphidium* has highly conserved structure, gene content and gene order, with little variation between species, based on complete chloroplast genome analysis (20 samples of 17 subg. *Seriphidium* species). Chloroplast genome size varied between the species, while there was sequence uniformity within species (Table 1). However, sequence variation has been reported within other species, such as *Ilex viridis* [45], *Calligonum junceum* [44] and *Calanthe davidii* [48]. Furthermore, this phenomenon was present in other subgenera of *Artemisia* [47, 49], such as *Artemisia selengensis*, *Artemisia argyi*, and *Artemisai annua*, however it is not found in subg. *Seriphidium*, probably due to the small sample size of the same species in the subgenus.

IR expansion and contraction is a common evolutionary phenomenon and often generates variation of chloroplast genome length [50]. Although the IR junctions of these subg. *Seriphidium* chloroplast genomes exhibited modest expansion or contraction (Fig. 2), the IR regions, which varied by 13 bp, were more conserved than the SC regions, which varied by 663 bp (for LSC regions) and 50 bp (for SSC regions) (Table 1). Moreover, IR expansion and contraction also play important roles in plastome rearrangements and gene content variations [50]. Although genome rearrangement has been reported for Compositae [51], Plantaginaceae [52] and Hypericaceae [53], this has not been observed in subg. *Seriphidium* (Additional file 2: Fig. S1, S4) and in other subgenera of *Artemisia* [21, 47, 49, 54].

### Repeated sequence analysis

As a result of their high rate of polymorphism and abundant variation at the species level, SSRs are commonly employed in genetic diversity, population structure and species classifications [55–57]. SSR distributions can be used to infer highly polymorphic regions, contributing to the development of molecular markers for inferring phylogenetic relationships [58]. Among the 1385 SSR loci identified in the 20 subg. *Seriphidium* chloroplast genomes (Additional file 1: Table S3), A/T motif mononucleotide repeats were abundant (Fig. S2d). This finding, which is consistent with similar pattern of SSRs distribution in chloroplast genomes of other subgenera in *Artemisia* and other genera in Asteraceae [21, 47, 49, 54, 58, 59], may be because polyA and polyT have more stable structures than polyC and polyG [60].

In closely related species, the abundant variation in long dispersed repeats longer than 30 bp provides some evolutionary flexibility [45]; further, it results in insertion/deletion mismatches and genome rearrangement [58]. Among the 818 long dispersed repeats in the 20 subg. *Seriphidium* chloroplast genomes (Additional file 1: Table S4), forward and palindromic repeats accounted for 398 (48.66%) and 394 (48.17%) of all repeats, respectively, while reverse and complementary repeats were quite rare, accounting for just 25 (3.05%) and 1 (0.12%), respectively. This pattern of long dispersed repeats is similar to other subgenera of *Artemisia* and other angiosperms [21, 40, 45, 47, 61–63].

### Hypervariable regions and molecular markers

Given that genes are not all equally important in the development of barcoding, or in population genetic and phylogenetic studies [21], screening of hypervariable regions can provide a wealth of phylogenetic information for such research [64–66]. We identified eight hypervariable regions, all within SC regions, with IR regions exhibiting lower variation (Fig. S3), consistent with our genomic divergence analysis (Additional file 2: Fig. S4). Phylogenetic analyses of *Artemisia* have often been based on plastid markers (mainly *matK*, *rbcL*, *trnL* – *trnF*, *psbA* – *trnH*, *rpl32* – *trnL* and *ndhC* – *trnV*), this has left many interspecific relationships poorly resolved, particularly in subg. *Seriphidium* [16, 17, 31]. When comparing these markers with the highly variable regions identified here, only two (*ndhC* – *trnV* and *rpl32* – *trnL*) have been used for phylogenetic inference in subg. *Seriphidium*, with weak resolution power [18]. Furthermore, the presence of rapid radiation differentiation in subg. *Seriphidium* has led to phylogenetic trees reconstructed based on either each highly variable regions screened or their tandem sequences being poorly resolved in terms of interspecific relationships (Additional file 2: Fig. S5–13). However, phylogenetic reconstructions of evolutionarily complex taxa using complete chloroplast genomes, such as those for *Calligonum* [44], *Hoya* [67] and *Ilex* [45], typically provide higher resolution and more stable backbones than those based on multiple gene fragments. Our results also confirmed that the whole chloroplast genome resolves interspecific relationships well in subg. *Seriphidium* (Additional file 2: Fig. S14), and the same effect was found in other subgenera of genus *Artemisia* [47, 61, 62]. This provides a good reference for using the whole chloroplast genome as superbarcodes to analysis the phylogenetic relationship of *Artemisia* and its allies.

### Phylogenetic inference

We have reconstructed the phylogenetic relationships of *Artemisia* via Bayesian inference and maximum likelihood, using 38 *Artemisia* species representing the most extensive chloroplast genome sample to date (Fig. 4). This work provides a solid and high-resolution phylogenetic backbone of *Artemisia*, revealing inconsistencies between molecular systematics and traditional taxonomic studies. Most of the morphologically derived subgenera and sections within *Artemisia* are revealed to be polyphyletic, suggesting that the morphologically derived classifications are inaccurate. To resolve the relationships within subg. *Seriphidium*, we sampled three major clades in this subgenus (Fig. 4). Our results validate the earlier molecular findings that merge the subg. *Seriphidium* into the genus *Artemisia* [4, 13, 15, 16, 18]. While some authors still consider *Seriphidium* to be an independent genus [17, 68], this view not supported by the current knowledge.

**Fig. 5:**
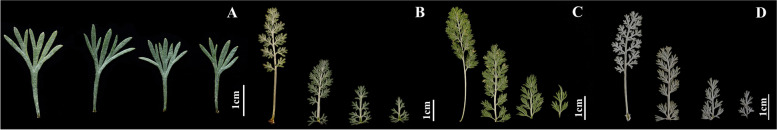
Leaves of four *Artemisia* subg. *Seriphidium* species. **A  ***Artemisia juncea* (sect. *Juncea*); **B** *Artemisia minchunensis* (sect. *Minchunensa*); **C ***Artemisia sawanensis* (sect. *Seriphidium*); **D ***Artemisia schrenkiana* (sect. *Seriphidium*). The sections of *Artemisia* subg. *Seriphidium* are divided according to Ling (1991) [19]

Here, subg. *Seriphidium* was revealed to be polyphyletic, divided into two clades separated by a large genetic distance, reaffirming previous molecular phylogenetic findings on subg. *Seriphidium* [18]. While various taxonomists have divided *A. juncea* into different sections or series within subg. *Seriphidium* based on morphology, none has been aware of its evolutionary differentiation extended beyond this subgenus boundaries [7, 19, 20, 26, 68]. According to our results of molecular systematics, the proposal of removing *A. juncea* from subg. *Seriphidium* to obtain a monophyletic subgenus [18] is supported. However, our observations on the morphological traits of *A. juncea* revealed that its bracts layer (4–5), homogamous bisexual florets (4–7) and leaf indumentum are consistent with the morphological characters of subg. *Seriphidium* taxa, but its palmately ternate leaf pattern is uncommon (Fig. 5A) in this subgenus. In view of this, the systematic position of *A. juncea* remains to be further explored by combining the evidence of morphology and molecular systematics.

Ling established *A. minchunensis* as a special group (sect. *Minchunensa*) within subg. *Seriphidium* mainly based on its leaves pectinately 2(or 3)-pinnatisect; lobules serrate or subserrate, arachnoid pubescent or glabrescent [19, 68]. The phylogenetic position of *A. minchunensis* has been unclear, owing to limited sampling in earlier molecular phylogenetic studies [18]. Here, our focused sampling revealed that *A. minchunensis* formed a highly supported (PP = 0.997; BS = 89) sister group to *A. sawanensis* and *A. schrenkiana* in sect. *Seriphidium*. Apparently our molecular phylogenetic results did not support the establishment of sect. *Minchunensa*. Actually, after careful observation of the leaf morphological characteristics of the above three species, we found that a high similarity in leaf morphology and indumentum, such as pinnatisect (bipartite or ternate) ovate or broadly ovate and densely pilose, with pinnately divided pseudo-stipules (Fig. 5B – D). Based on our molecular phylogenetic studies and morphological observations, it is considered inappropriate to establish morphologically-based sect. *Minchunensa*, which should be abolished and placed within sect. *Seriphidium*.

## Conclusions

We newly sequenced 18 chloroplast genomes of 16 subg. *Seriphidium* species and compared them with one previously published taxon. Comparative analysis showed that genomic structures and gene order were relatively conserved, with only some variation in IR borders. Phylogenetic analysis revealed inconsistencies between the molecular phylogeny and traditional taxonomy of the subg. *Seriphidium* and the whole chloroplast genomes can be used as superbarcodes to resolve interspecific relationships in this subgenus. In future, combining complete chloroplast genomes and morphological data, based on detailed sampling, could enhance our understanding of the complex phylogenetic relationships in this group, providing the basis for a worldwide taxonomic revision of *Artemisia* subg. *Seriphidium*.

## Materials and methods

### Taxon sampling, DNA extraction, and sequencing

In total, 18 samples of 16 *Artemisia* subg. *Seriphidium* species were collected from northwestern China and adjacent countries (Russia and Tajikistan). For most of the species in the subgenus, we sampled one individuals, except for *A. minchunensis* and *A. juncea*, for which we sampled two individuals each (Table 1). No specific permissions were required for our locations/activities. Additional file 1 (Table S1) provides GenBank information for the remaining species used in the phylogenetic analysis. Nomenclature follows the accepted World Flora Online (http://www.worldfloraonline.org/) species names for the subg. *Seriphidium*. Voucher specimens were deposited in the Herbarium of the Xinjiang Institute of Ecology and Geography Chinese Academy of Sciences (XJBI) and the Herbarium of the Institute of Botany, Chinese Academy of Sciences (PE).

Total genomic DNA was extracted from ca. 100 mg of silica-dried leaves and isolated according to the cetyltrimethyl ammonium bromide (CTAB) method [69]. Extracted DNA samples were randomly fragmented to construct a 300 bp short-insert library and − 2 × 150 bp paired-end (PE) reads were performed on DNBSEQ™ technology platforms at the Beijing Genomics Institute (BGI, Shenzhen, China). The raw reads were evaluated using fastQC 0.11.5 (http://www.bioinformatics.babraham.ac.uk/projects/fastqc/), and edited using Trimmomatic 0.35 [70] to remove adapters and low-quality bases. Finally ca. 2.5 G bp paired-end clean read was obtained for each sample.

### Chloroplast genome assembly and annotation

The clean data were assembled using GetOrganelle v. 1.7.1 [71], The complete circular assembly graph was checked and further extracted using Bandage v. 0.8.1 [72]. The finished plastid genomes were annotated by DOGMA [73], and GeSeq [74], and then manually adjusted by Geneious v. 9.1.7 [75]. Gene start and stop codons were determined via comparison with the *A. maritima* (NC_045093) and *A. annua* (NC_034683) genomes. The annotated plastid genomes were submitted to GenBank (Table 1) and Organellar Genome Draw (OGDRAW) [76] was used to illustrate a circular genome map.

### Genome comparison and divergence analysis

Sequence alignment of the 20 subg. *Seriphidium* samples complete chloroplast genomes was conducted using MAFFT v. 7 [77]. The Mauve v. 2.3.1 [78], with default parameters, was used to identify locally collinear blocks among the chloroplast genomes. The genome variability across the 20 subg. *Seriphidium* samples was assessed using mVISTA [79] in Shuffle-LAGAN mode. Expansions and contractions of inverted repeat regions were visualized at the junctions of the four main (LSC/IRb/SSC/IRa) of the chloroplast genome, via IRScope [80]. Nucleotide diversity (Pi) was estimated by sliding window analysis conducted in DnaSP v. 6 [81] (window length, 600 bp; step size, 200 bp).

### Repetitive sequences analysis

Simple sequence repeats (SSRs) across the 20 plastomes were identified using web-MISA [82] with the following parameters: ten repetitions for mononucleotide motifs, five for dinucleotide motifs, four for trinucleotide motifs and three for tetranucleotide, pentanucleotide and hexanucleotide motifs. The long dispersed repeats (LDRs): including forward (F), palindromic (P), reverse (R), and complement (C) repeats were identified using the online tool REPuter [83], with a Hamming distance of 3 and a minimum repeat size of 30 bp.

### Phylogenetic analyses

Phylogenetic analyses were conducted using 80 protein-coding genes and 52 complete chloroplast genomes (after removing one inverted repeat). In total 38 *Artemisia* species from four subgenera and 10 sections, including 17 subg. *Seriphidium* species from three sections, were used for phylogenetic analysis (Fig. 4). *Ajania pacifica* (Accessions NC_050690 and MN883841) was used as the outgroup. Genome alignment was performed by MAFFT v. 7 [77] and trimmed using the “-gappyout” setting in trimAI v. 1.2, a PhyloSuite [84] plugin. According to the Bayesian information criterion (BIC), the most appropriate substitution models, estimated using jModelTest2 [85], were TVM + I + G for the complete chloroplast genome sequences and the protein-coding genes. Maximum likelihood (ML) analyses were conducted using RaxML-HPC v.8 [86], with 1000 bootstrap iterations. Based on the eight hypervariable regions screened and their tandem sequences, using ML method to reconstruct phylogenetic tree respectively in accordance with the above method. Only first the eight hypervariable regions screened were manually extracted and concatenated from the whole chloroplast genomes of 17 subg. *Seriphidium* species (16 newly sequenced and one published) by Geneious v. 9.1.7 [75]. Bayesian inference (BI) analysis was carried out using MrBayes v.3.2 [87], with Markov chain Monte Carlo simulations algorithm (MCMC) for 2,000,000,000 generations, using four incrementally-heated chains. This was conducted on the CIPRES Science Gateway portal [88]. The final trees were visualized and edited using FigTree v. 1.4.2 [89].

## Supplementary Information

**Additional file 1: Table S1.** GenBank information for species derived from the NCBI database used in the phylogenetic analysis. **Table S2.** List of annotated genes in the subg. *Seriphidium* chloroplast genomes. **Table S3.** Raw data from the analysis of simple sequence repeats in the subg. *Seriphidium*. **Table S4.** Raw data from the analysis of long dispersed repeats in the subg. *Seriphidium*. **Table S5.** Raw values for each variant region of the subg. *Seriphidium* chloroplast genome used for hypervariable regions analysis.

**Additional file 2: Figure S1.** Intraspecific synteny analyses of 20 subg. *Seriphidium* chloroplast genomes. The *A. ferganensis* chloroplast genome appears at the top as the reference sequence. Within each of the Mauve alignments, locally collinear blocks are indicated the same color and are connected by lines. **Figure S2.** Analysis of simple sequence repeats (SSRs) of the 20 *Artemisia* subg. *Seriphidium* chloroplast genomes. a. Numbers of the six SSR types; b. Numbers of SSRs distributed in the various copy regions; c. NumberS of SSRs distributed in various gene regions; d. Numbers of SSR repeat unit types. **Figure S3.** Sliding-window analysis of nucleotide diversity (Pi) of the aligned *Artemisia* subg. *Seriphidium* chloroplast genomes (window length 800 bp; step size 200 bp). **Figure S4.** Variation in subg. *Seriphidium* chloroplast genome sequences. Y axis: variation (50–100%). X axis: coordinate in the chloroplast genome. **Figure S5.** Phylogenetic tree constructed using the maximum likelihood method based on highly variable sequences (*ndhC* – *trnV-UAC*) selected from 17 subg. *Seriphidium* species (16 newly sequenced and one published). Numbers near the nodes is maximum likelihood bootstrap support values. **Figure S6.** Phylogenetic tree constructed using the maximum likelihood method based on highly variable sequences (*ndhF*) selected from 17 subg. *Seriphidium* species (16 newly sequenced and one published). Numbers near the nodes is maximum likelihood bootstrap support values. **Figure S7.** Phylogenetic tree constructed using the maximum likelihood method based on highly variable sequences (*ndhG – ndhI*) selected from 17 subg. *Seriphidium* species (16 newly sequenced and one published). Numbers near the nodes is maximum likelihood bootstrap support values. **Figure S8.** Phylogenetic tree constructed using the maximum likelihood method based on highly variable sequences (*rpl32 – trnL-UAG*) selected from 17 subg. *Seriphidium* species (16 newly sequenced and one published). Numbers near the nodes is maximum likelihood bootstrap support values. **Figure S9.** Phylogenetic tree constructed using the maximum likelihood method based on highly variable sequences (*trnE-UUC – ropB*) selected from 17 subg. *Seriphidium* species (16 newly sequenced and one published). Numbers near the nodes is maximum likelihood bootstrap support values. **Figure S10.** Phylogenetic tree constructed using the maximum likelihood method based on highly variable sequences (*trnK-UUU – rps16*) selected from 17 subg. *Seriphidium* species (16 newly sequenced and one published). Numbers near the nodes is maximum likelihood bootstrap support values. **Figure S11.** Phylogenetic tree constructed using the maximum likelihood method based on highly variable sequences (*trnT-GGU*) selected from 17 subg. *Seriphidium* species (16 newly sequenced and one published). Numbers near the nodes is maximum likelihood bootstrap support values. **Figure S12.** Phylogenetic tree constructed using the maximum likelihood method based on highly variable sequences (*ycf1*) selected from 17 subg. *Seriphidium* species (16 newly sequenced and one published). Numbers near the nodes is maximum likelihood bootstrap support values. **Figure S13.** Phylogenetic tree constructed using the maximum likelihood method based on tandem sequences from eight highly variable regions selected from 17 subg. *Seriphidium* species (16 newly sequenced and one published). Numbers near the nodes is maximum likelihood bootstrap support values. **Figure S14.** Phylogenetic tree constructed using the maximum likelihood method based on the whole chloroplast genomes of 17 subg. *Seriphidium* species (16 newly sequenced and one published). Numbers near the nodes is maximum likelihood bootstrap support values.

## Data Availability

All the newly sequenced sequences in this study are available from the National Center for Biotechnology Information (NCBI) (https://www.ncbi.nlm.nih.gov/; accession numbers: ON871797 – ON871813 and OL890688; see Table 1). Information for other samples used for phylogenetic analysis download from GenBank can be found in Additional file 1: Table S1.

## Abbreviations

cp: Chloroplast
CDS: Coding sequence
IRs: Inverted repeat regions
IRa, IRb: Two IR regions that are identical but in opposite orientations
LSC: Large single copy
SSC: Small single copy
GC: Guanine-cytosine
tRNA: Transfer RNA
rRNA: Ribosomal RNA
SSRs: Simple sequence repeats
LDRs: Long dispersed repeats
IGS: Intergenic regions
ITS: Nuclear ribosomal internal transcribed space
ETS: Nuclear ribosomal external transcribed space
Pi: Nucleotide diversity
CTAB: Cetyltrimethyl ammonium bromide method
DnaSP: DNA Sequences Polymorphism
DOGMA: Dual Organellar Genome Annotator
MCMC: Markov Chain Monte Carlo
BIC: Bayesian information criterion
ML: Maximum Likelihood
PP: Posterior probability
BI: Bayesian Inference
BS: Bootstrap
NCBI: National Center for Biotechnology Information
