# Supplementary material for: Comparative analysis of complete Artemisia subgenus Seriphidium (Asteraceae: Anthemideae) chloroplast genomes: insights into structural divergence and phylogenetic relationships
Source: BMC Plant Biol. 2023 Mar 10;23:136. doi: 10.1186/s12870-023-04113-1 (PMC9999589; doi:10.1186/s12870-023-04113-1)
Supplement: Supplementary file 2 — Additional file 2: Figure S1. Intraspecific synteny analyses of 20 subg. Seriphidium chloroplast genomes. The A. ferganensis chloroplast genome appears at the top as the reference sequence. Within each of the Mauve alignments, locally collinear blocks are indicated the same color and are connected by lines. Figure S2. Analysis of simple sequence repeats (SSRs) of the 20 Artemisia subg. Seriphidium chloroplast genomes. a. Numbers of the six SSR types; b. Numbers of SSRs distributed in the various copy regions; c. NumberS of SSRs distributed in various gene regions; d. Numbers of SSR repeat unit types. Figure S3. Sliding-window analysis of nucleotide diversity (Pi) of the aligned Artemisia subg. Seriphidium chloroplast genomes (window length 800 bp; step size 200 bp). Figure S4. Variation in subg. Seriphidium chloroplast genome sequences. Y axis: variation (50–100%). X axis: coordinate in the chloroplast genome. Figure S5. Phylogenetic tree constructed using the maximum likelihood method based on highly variable sequences (ndhC – trnV-UAC) selected from 17 subg. Seriphidium species (16 newly sequenced and one published). Numbers near the nodes is maximum likelihood bootstrap support values. Figure S6. Phylogenetic tree constructed using the maximum likelihood method based on highly variable sequences (ndhF) selected from 17 subg. Seriphidium species (16 newly sequenced and one published). Numbers near the nodes is maximum likelihood bootstrap support values. Figure S7. Phylogenetic tree constructed using the maximum likelihood method based on highly variable sequences (ndhG – ndhI) selected from 17 subg. Seriphidium species (16 newly sequenced and one published). Numbers near the nodes is maximum likelihood bootstrap support values. Figure S8. Phylogenetic tree constructed using the maximum likelihood method based on highly variable sequences (rpl32 – trnL-UAG) selected from 17 subg. Seriphidium species (16 newly sequenced and one published). Numbers near the nodes is [file 12870_2023_4113_MOESM2_ESM.docx]

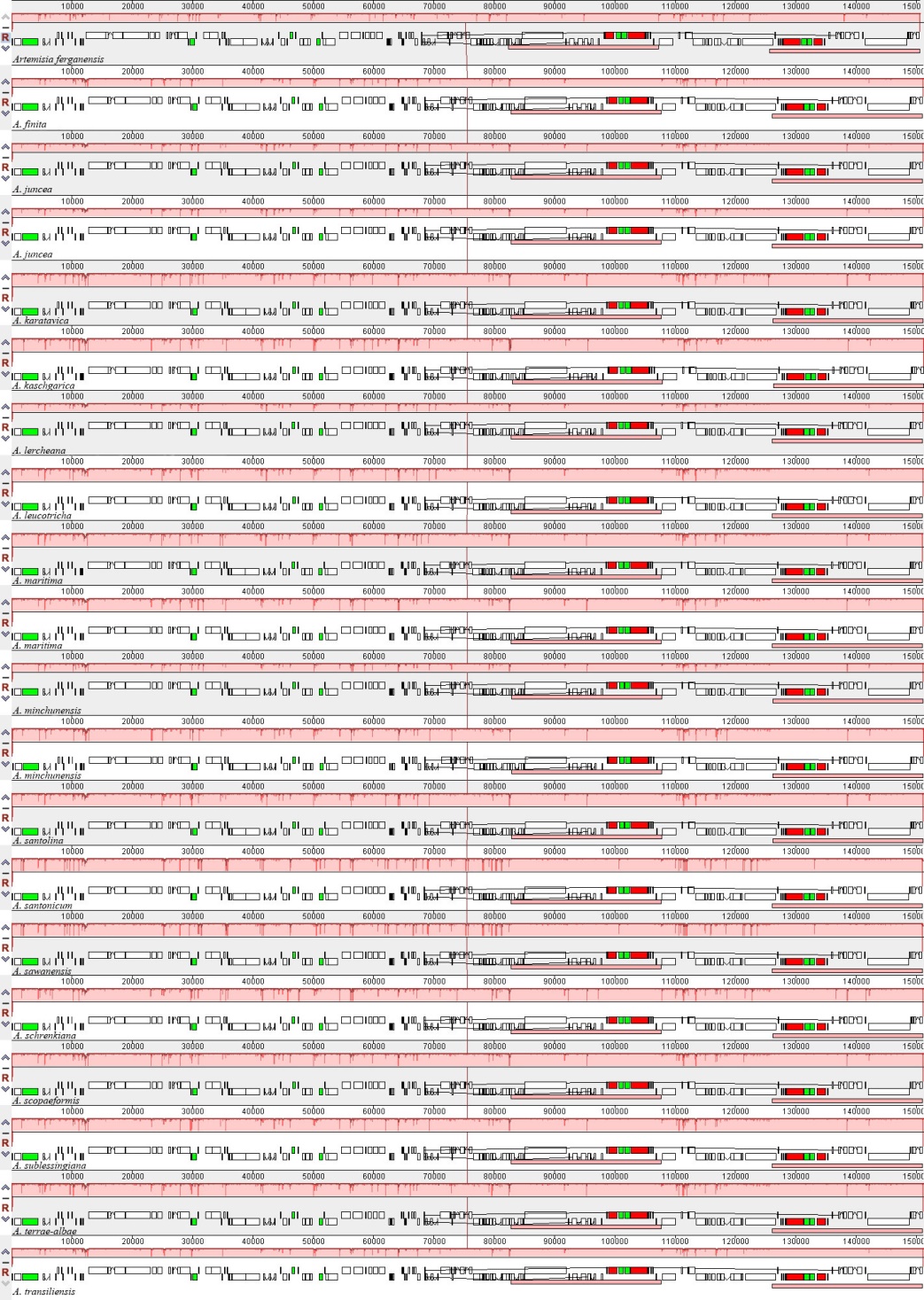


**Figure S1.** Intraspecific synteny analyses of 20 subg. *Seriphidium* chloroplast genomes. The *A. ferganensis* chloroplast genome appears at the top as the reference sequence. Within each of the Mauve alignments, locally collinear blocks are indicated the same color and are connected by lines.


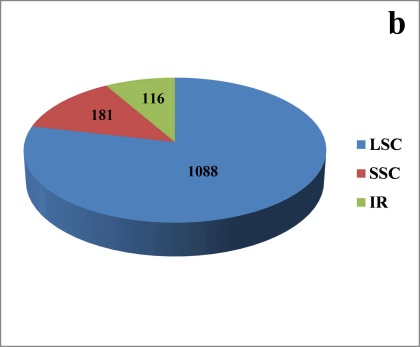

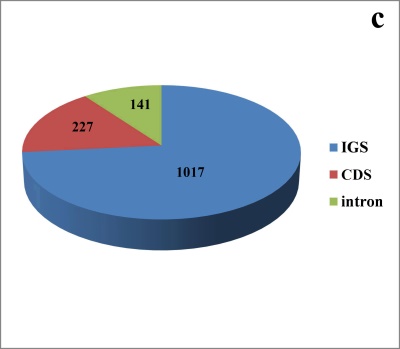


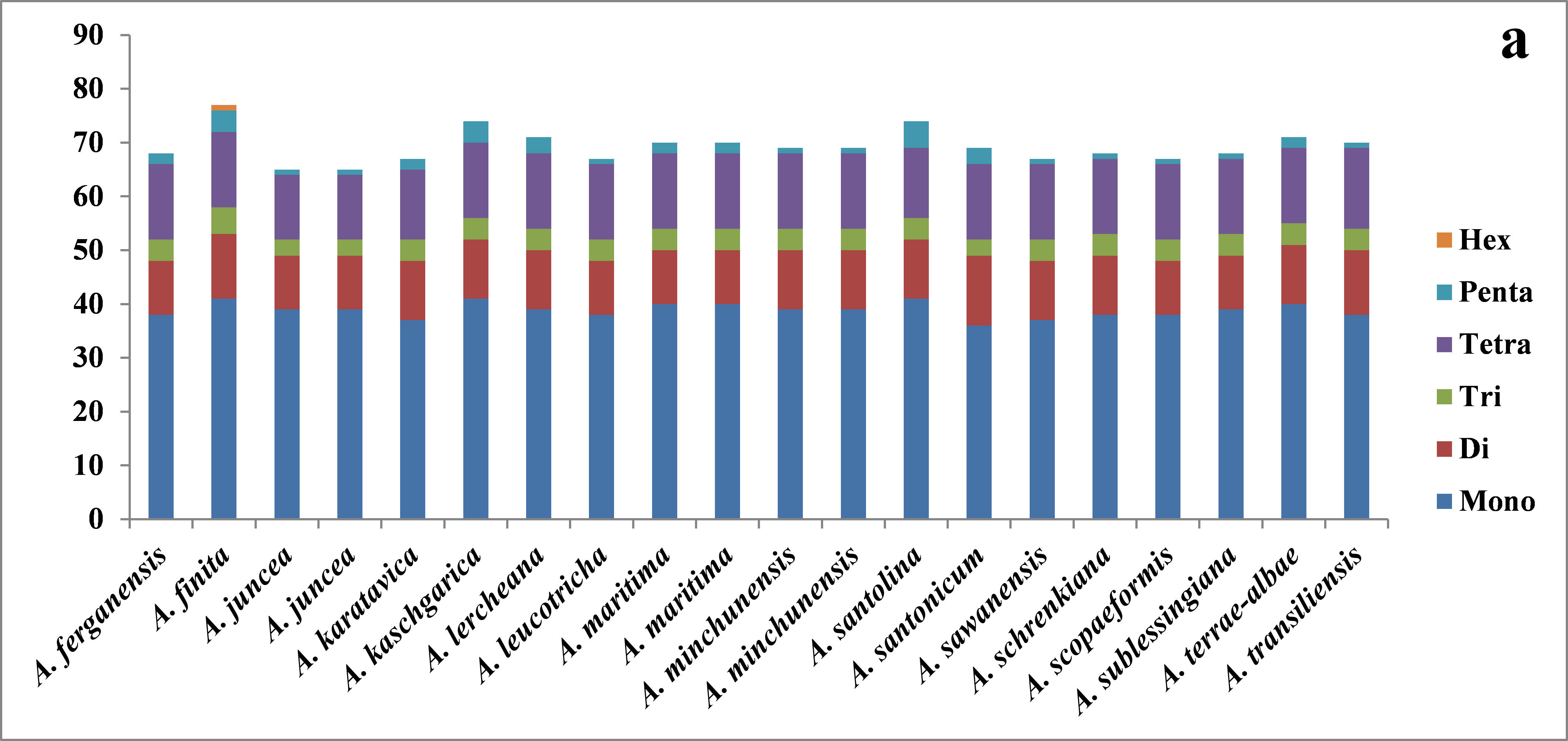

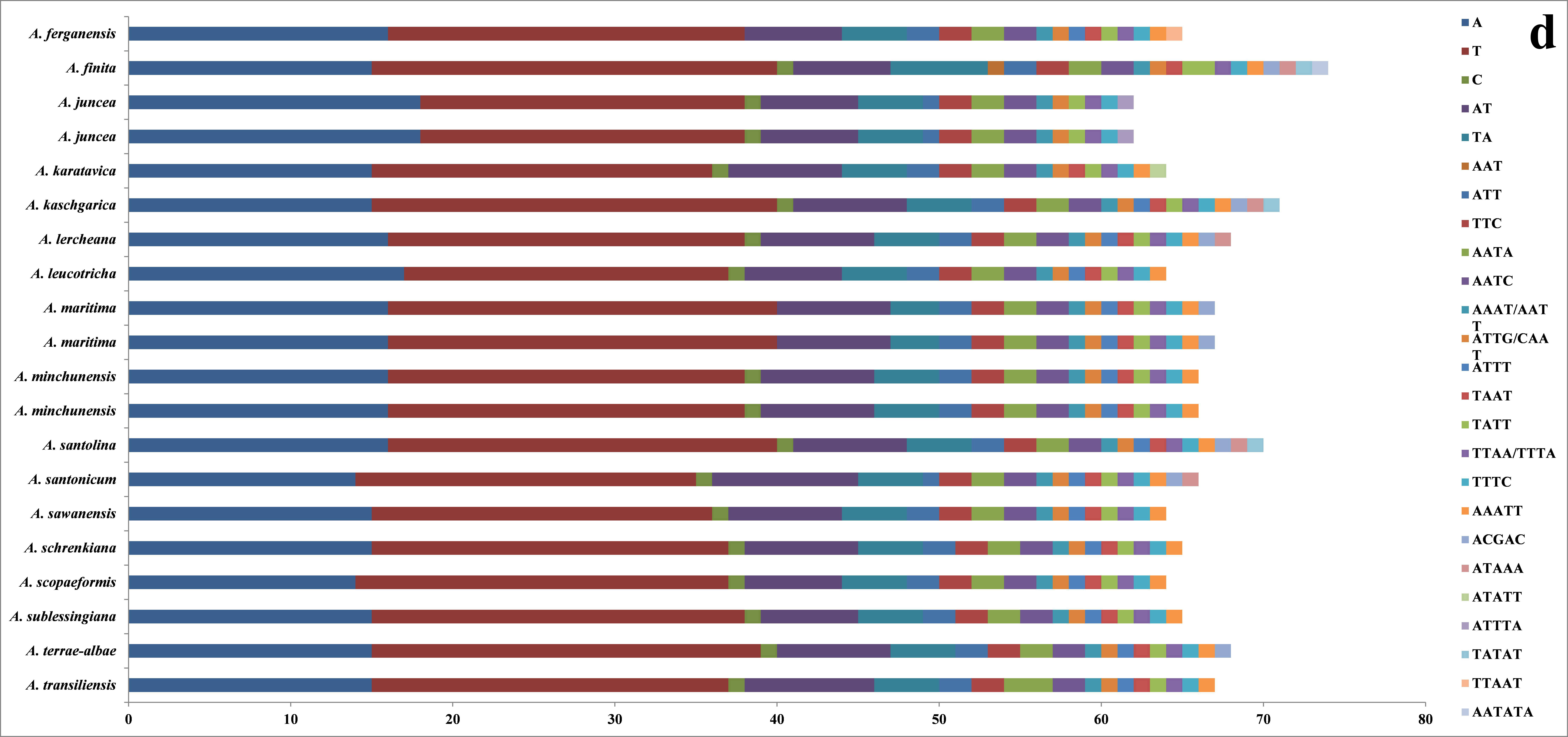


**Figure S2.** Analysis of simple sequence repeats (SSRs) of the 20 *Artemisia* subg. *Seriphidium* chloroplast genomes. **a.** Numbers of the six SSR types; **b.** Numbers of SSRs distributed in the various copy regions; **c.** NumberS of SSRs distributed in various gene regions; **d.** Numbers of SSR repeat unit types.


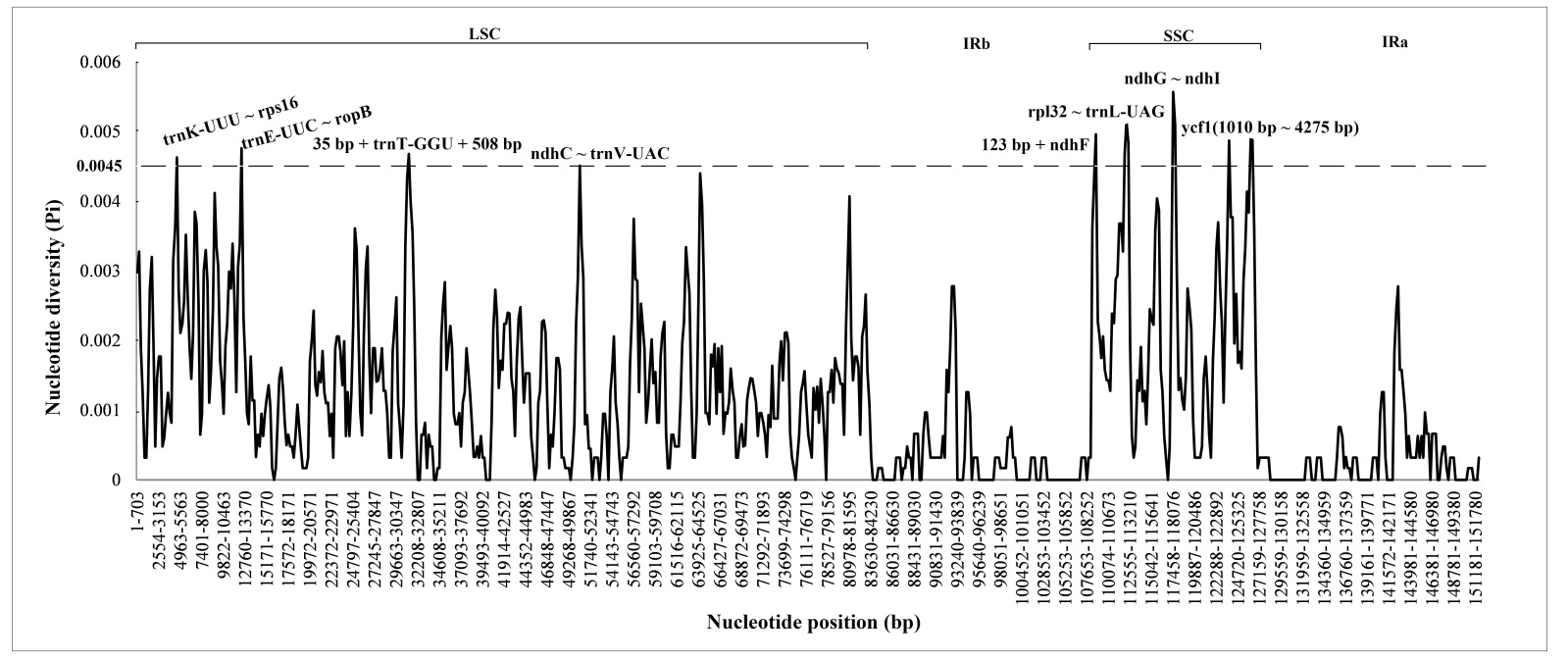


**Figure S3.** Sliding-window analysis of nucleotide diversity (Pi) of the aligned *Artemisia* subg. *Seriphidium* chloroplast genomes (window length 800 bp; step size 200 bp).


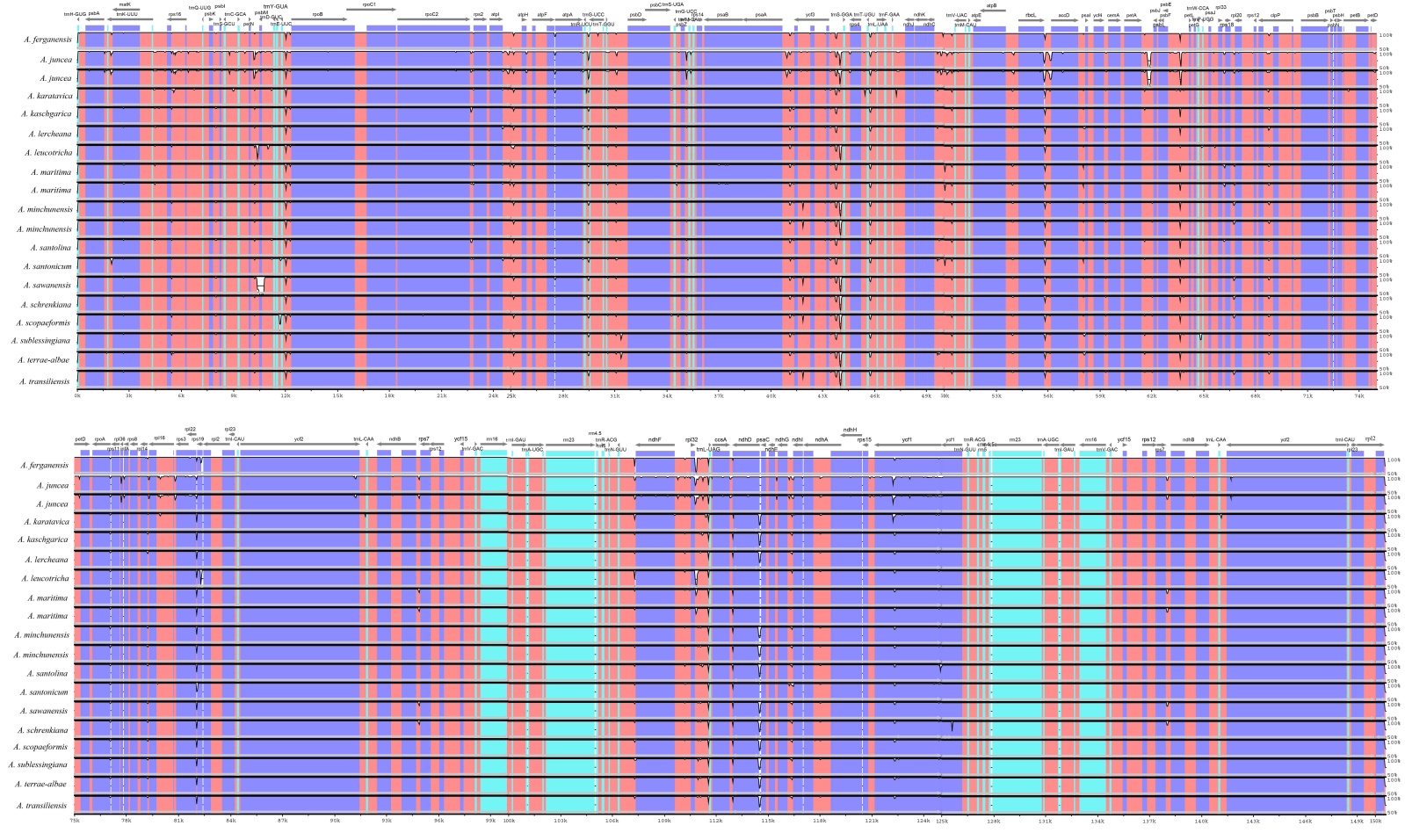


**Figure S4.** Variation in subg. *Seriphidium* chloroplast genome sequences. Y axis: variation (50 – 100%). X axis: coordinate in the chloroplast genome.


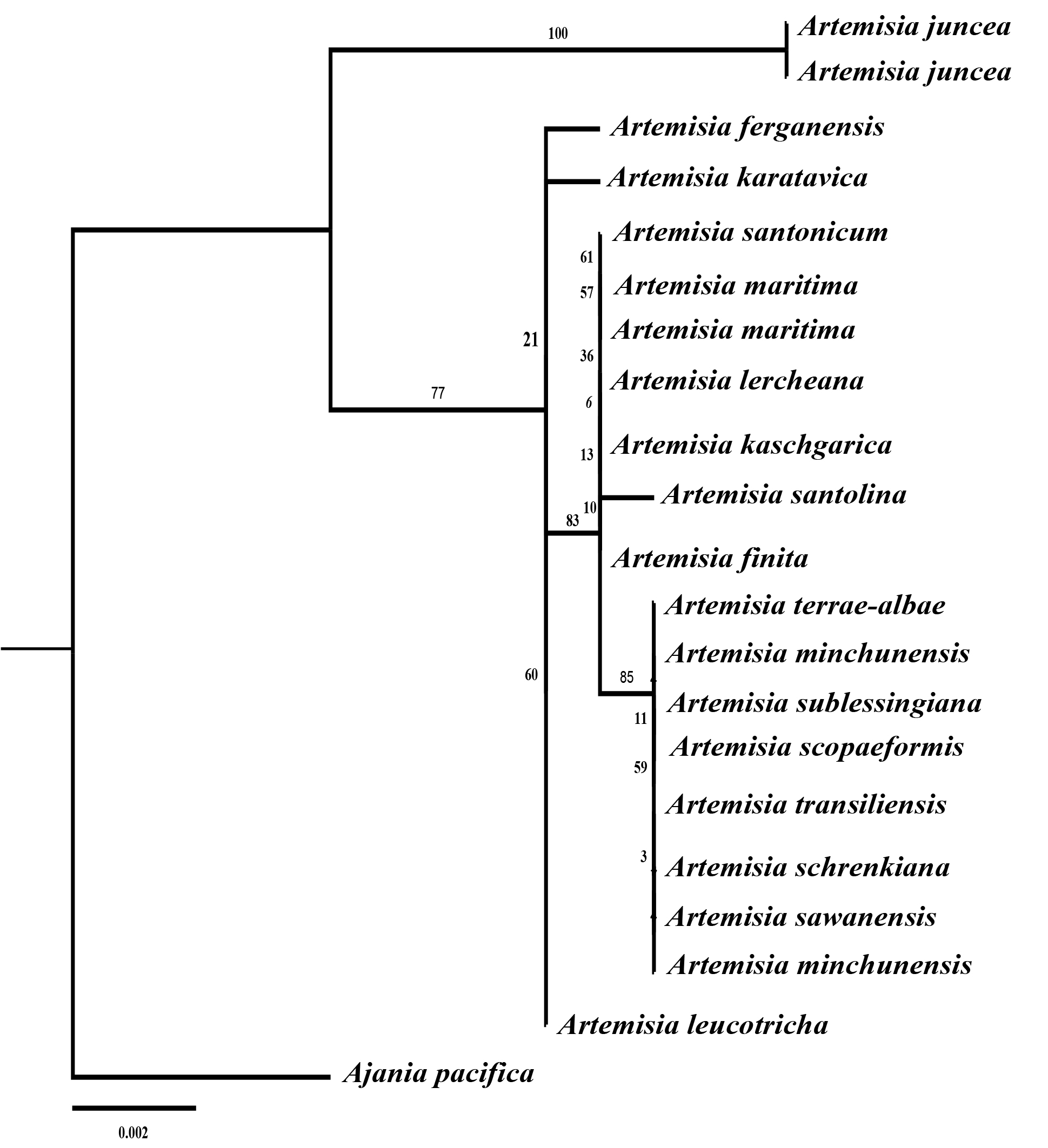


**Figure S5.** Phylogenetic tree constructed using the maximum likelihood method based on highly variable sequences (*ndhC* – *trnV-UAC*) selected from 17 subg. *Seriphidium* species (16 newly sequenced and one published). Numbers near the nodes is maximum likelihood bootstrap support values.


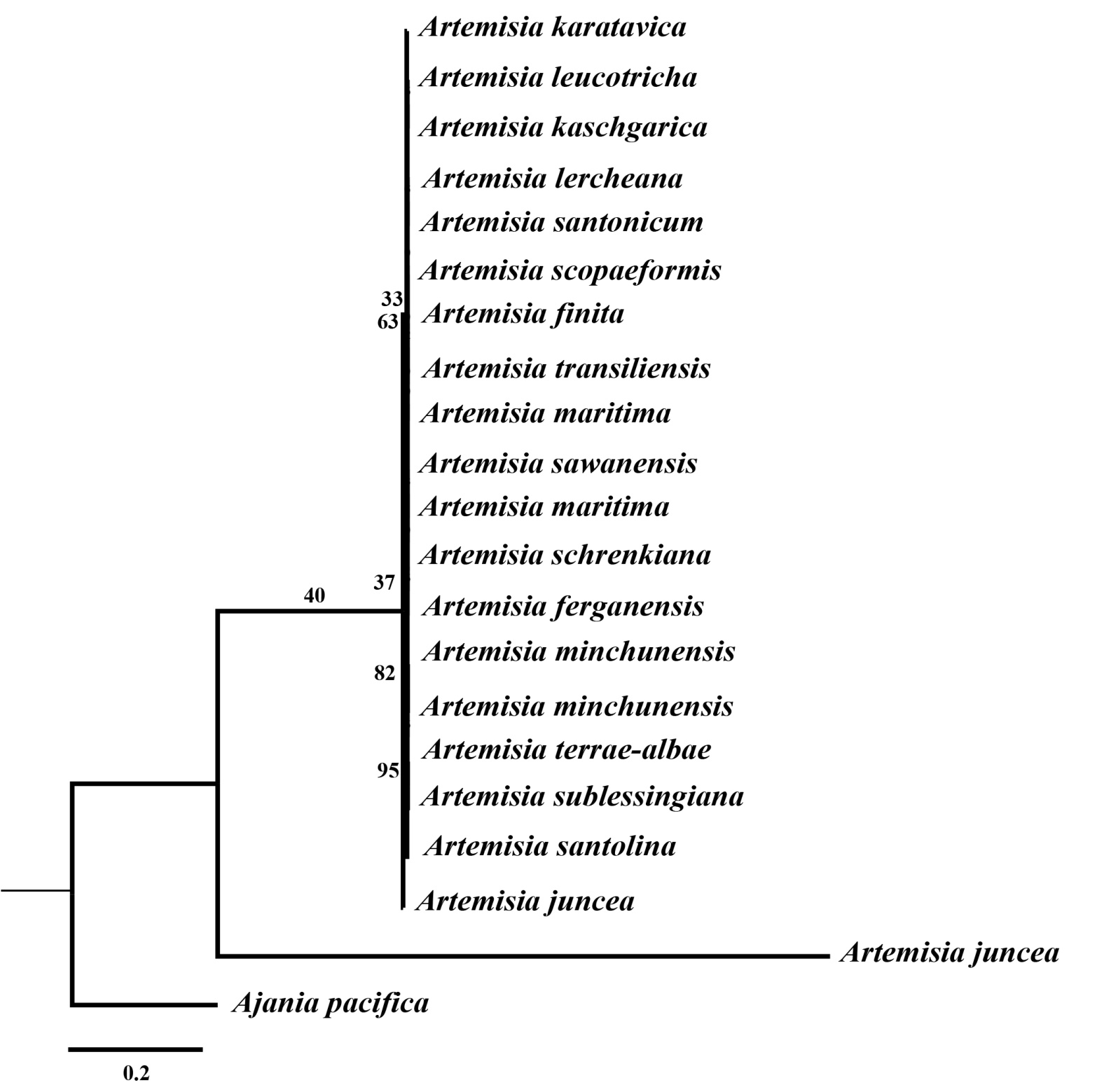


**Figure S6.** Phylogenetic tree constructed using the maximum likelihood method based on highly variable sequences (*ndhF*) selected from 17 subg. *Seriphidium* species (16 newly sequenced and one published). Numbers near the nodes is maximum likelihood bootstrap support values.


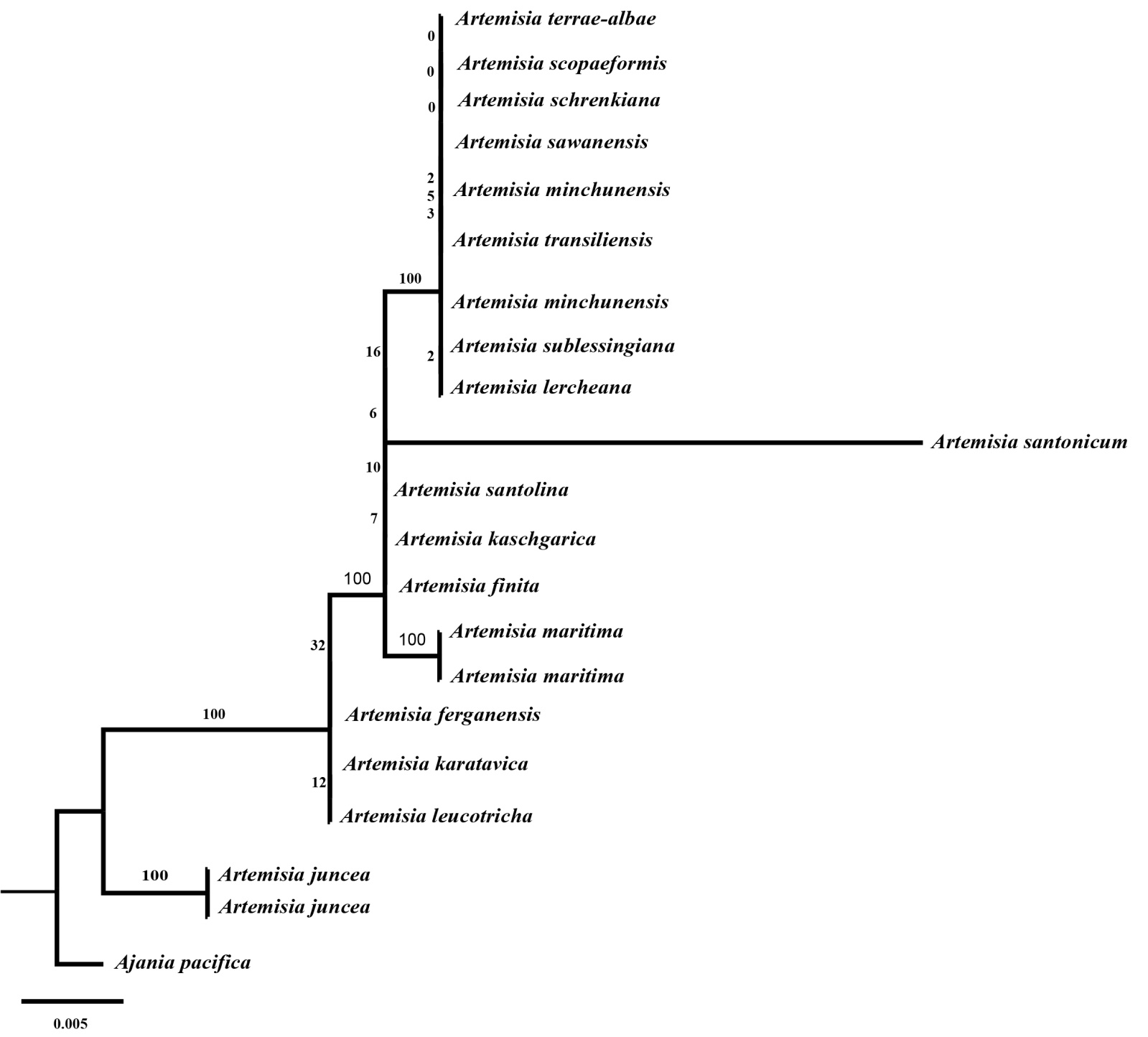


**Figure S7.** Phylogenetic tree constructed using the maximum likelihood method based on highly variable sequences (*ndhG – ndhI*) selected from 17 subg. *Seriphidium* species (16 newly sequenced and one published). Numbers near the nodes is maximum likelihood bootstrap support values.


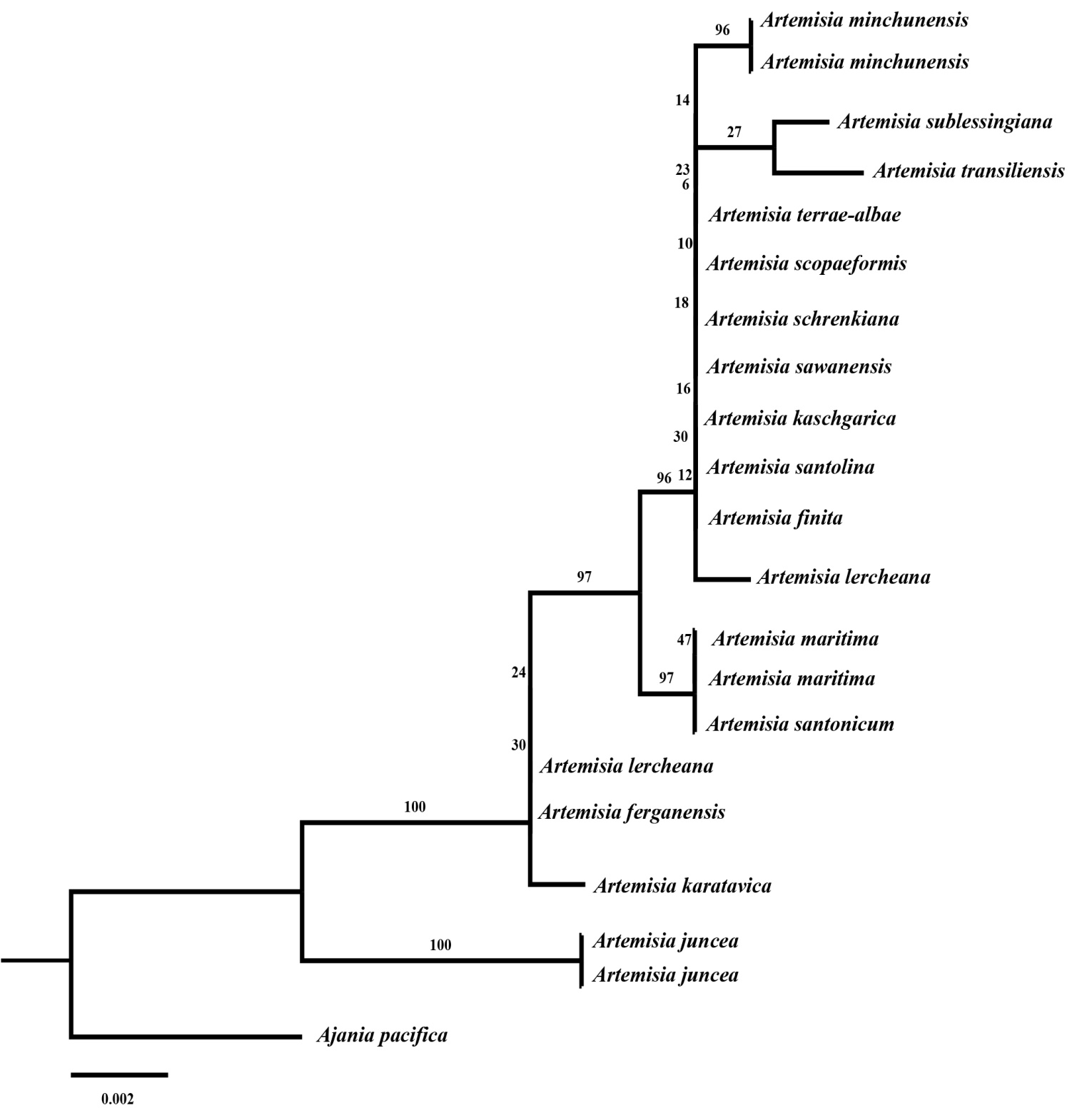


**Figure S8.** Phylogenetic tree constructed using the maximum likelihood method based on highly variable sequences (*rpl32 – trnL-UAG*) selected from 17 subg. *Seriphidium* species (16 newly sequenced and one published). Numbers near the nodes is maximum likelihood bootstrap support values.


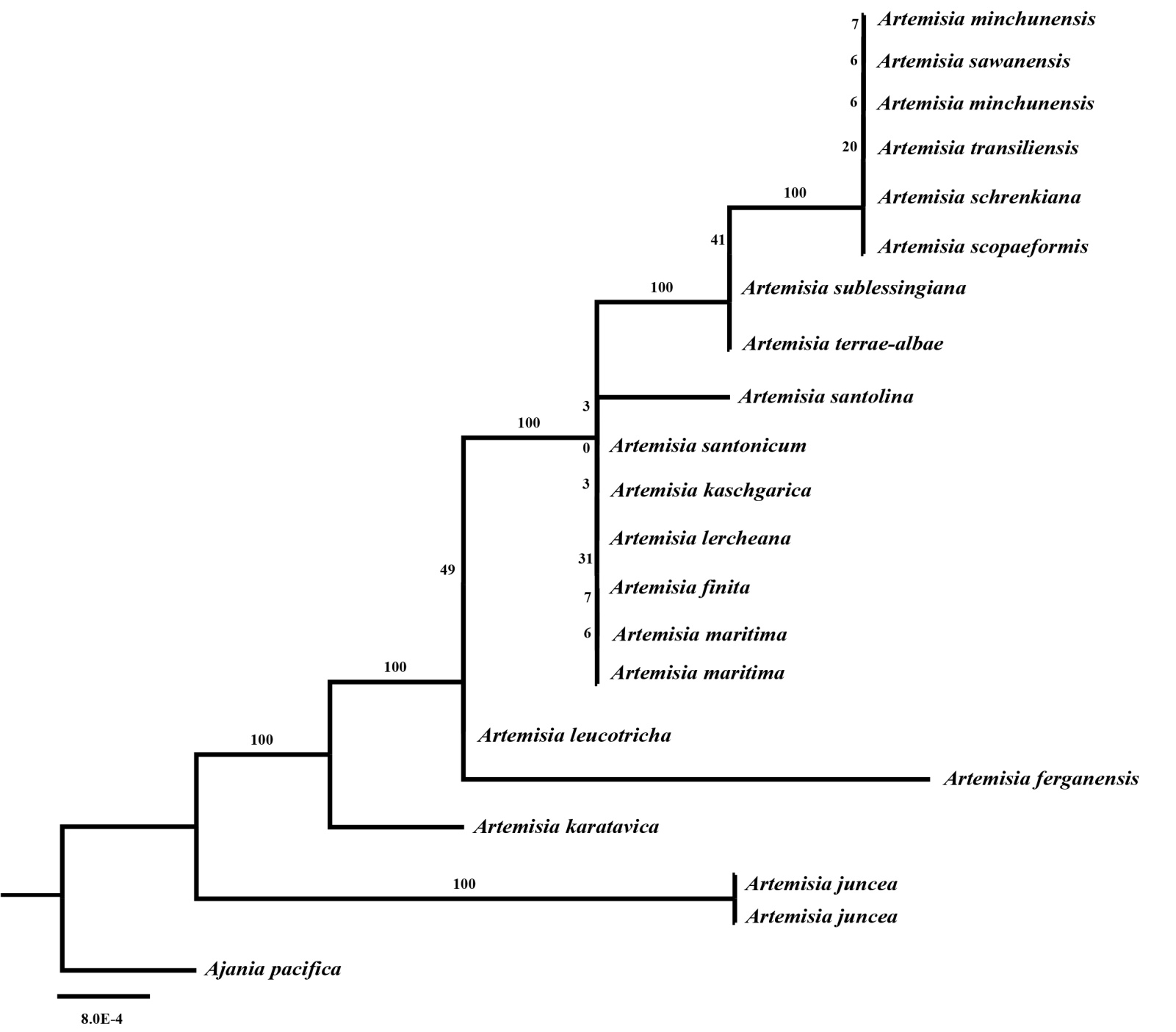


**Figure S9.** Phylogenetic tree constructed using the maximum likelihood method based on highly variable sequences (*trnE-UUC – ropB*) selected from 17 subg. *Seriphidium* species (16 newly sequenced and one published). Numbers near the nodes is maximum likelihood bootstrap support values.


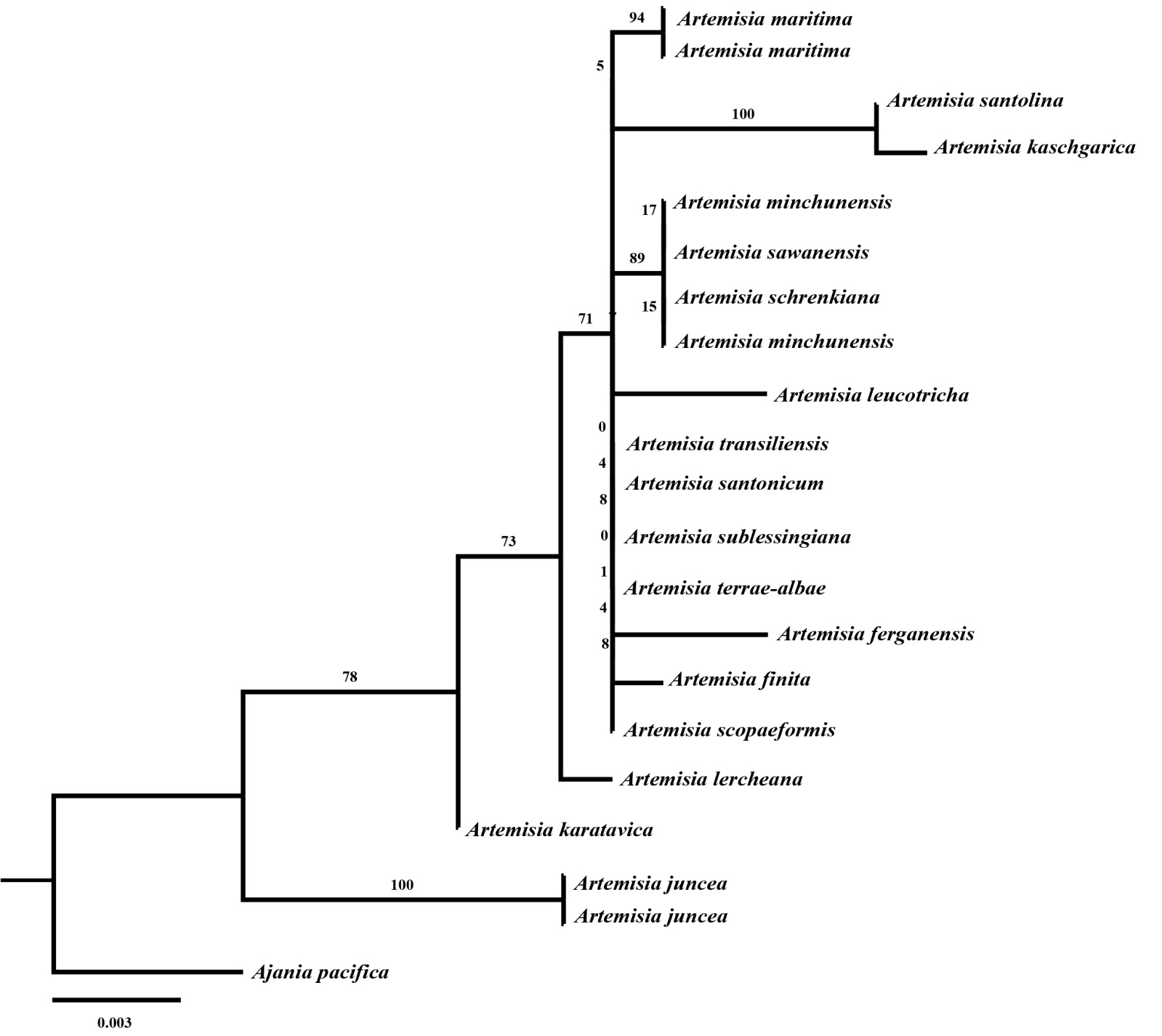


**Figure S10.** Phylogenetic tree constructed using the maximum likelihood method based on highly variable sequences (*trnK-UUU – rps16*) selected from 17 subg. *Seriphidium* species (16 newly sequenced and one published). Numbers near the nodes is maximum likelihood bootstrap support values.


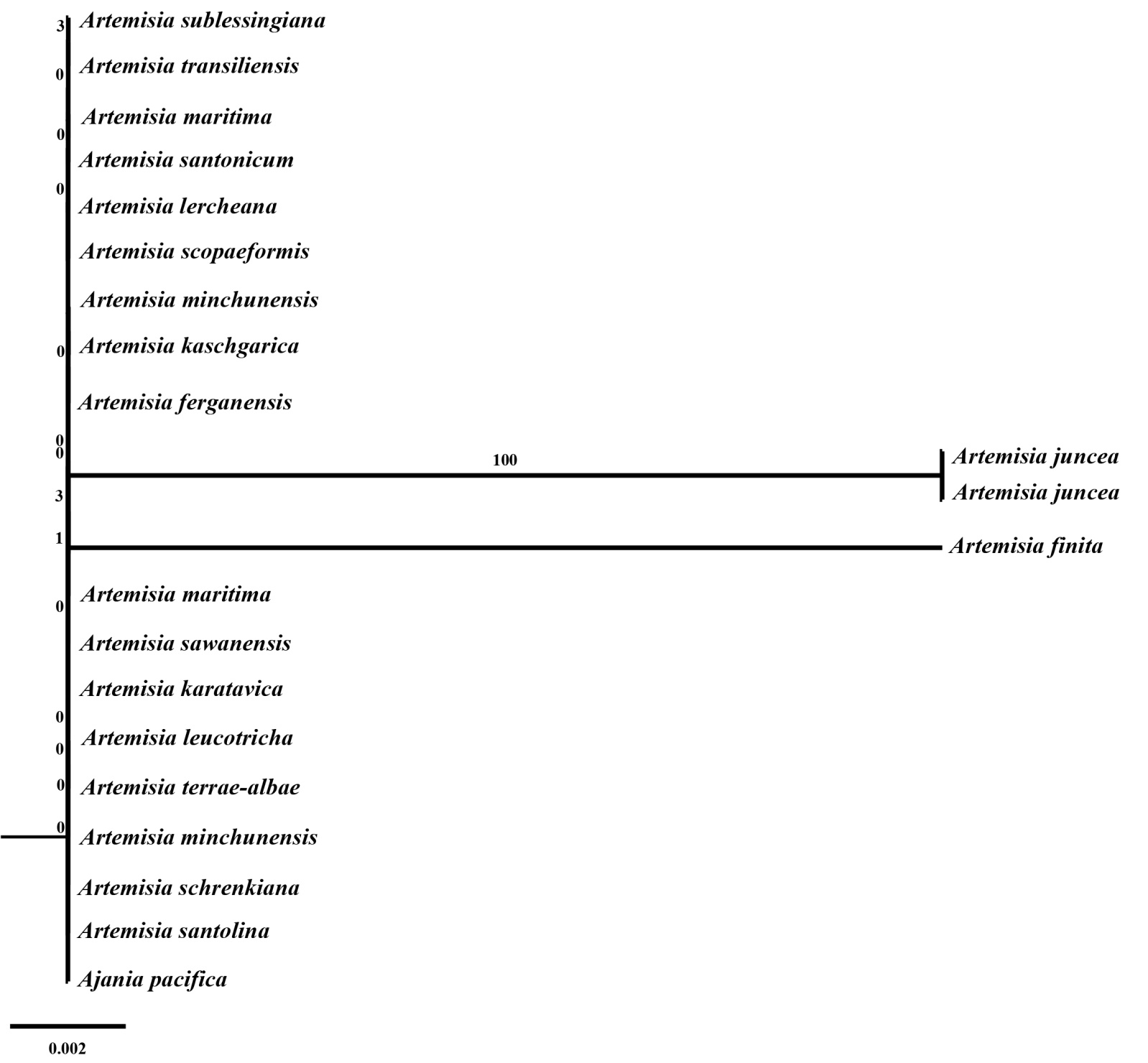


**Figure S11.** Phylogenetic tree constructed using the maximum likelihood method based on highly variable sequences (*trnT-GGU*) selected from 17 subg. *Seriphidium* species (16 newly sequenced and one published). Numbers near the nodes is maximum likelihood bootstrap support values.


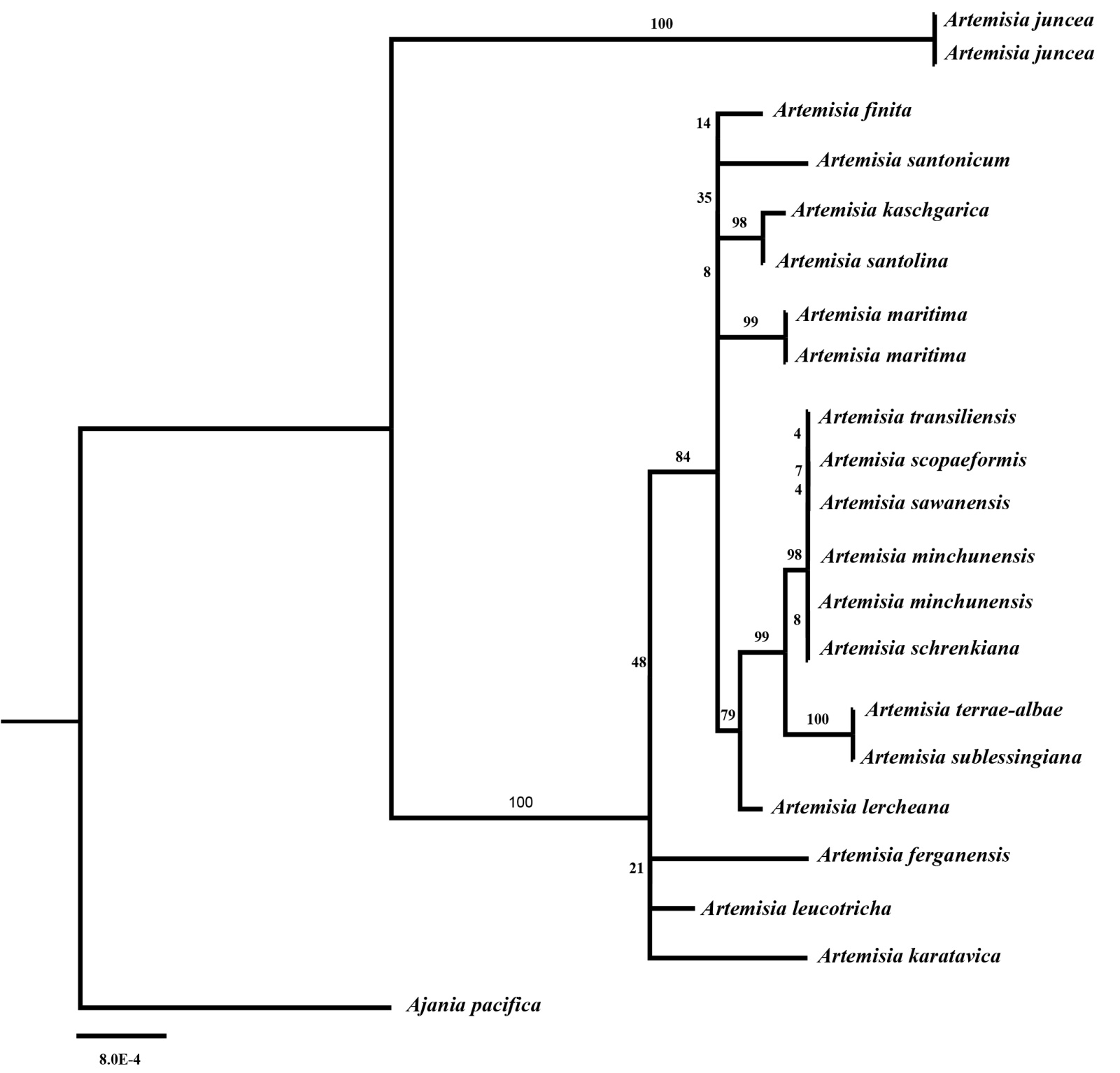


**Figure S12.** Phylogenetic tree constructed using the maximum likelihood method based on highly variable sequences (*ycf1*) selected from 17 subg. *Seriphidium* species (16 newly sequenced and one published). Numbers near the nodes is maximum likelihood bootstrap support values.


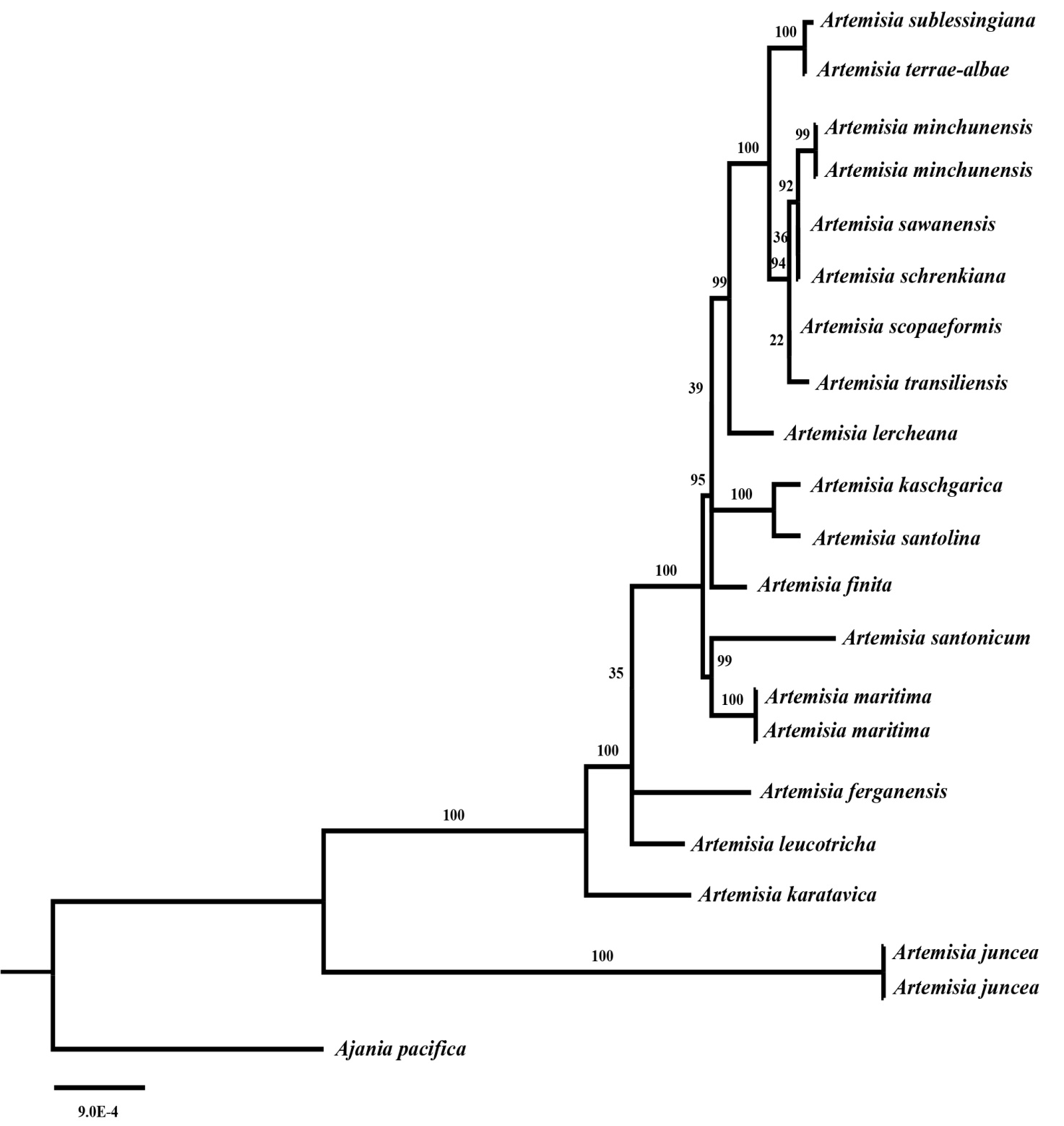


**Figure S13.** Phylogenetic tree constructed using the maximum likelihood method based on tandem sequences from eight highly variable regions selected from 17 subg. *Seriphidium* species (16 newly sequenced and one published). Numbers near the nodes is maximum likelihood bootstrap support values.


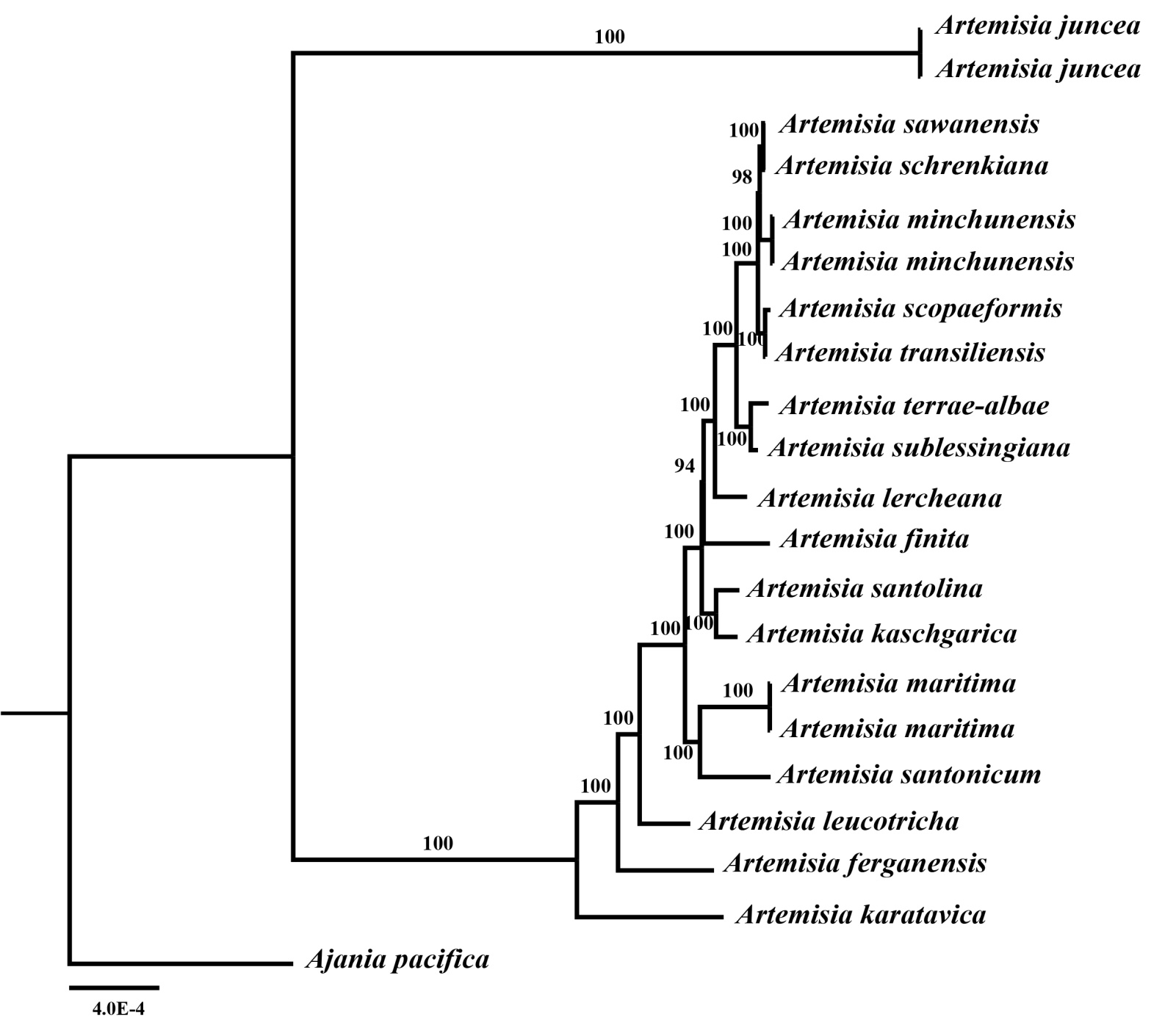


**Figure S14.** Phylogenetic tree constructed using the maximum likelihood method based on the whole chloroplast genomes of 17 subg. *Seriphidium* species (16 newly sequenced and one published). Numbers near the nodes is maximum likelihood bootstrap support values.
